# Primer-free FISH probes from metagenomics/metatranscriptomics data permit the study of uncharacterised taxa in complex microbial communities

**DOI:** 10.1038/s41522-019-0090-9

**Published:** 2019-06-25

**Authors:** Shi Ming Tan, Pui Yi Maria Yung, Paul E. Hutchinson, Chao Xie, Guo Hui Teo, Muhammad Hafiz Ismail, Daniela I. Drautz-Moses, Peter F. R Little, Rohan B. H. Williams, Yehuda Cohen

**Affiliations:** 1 Interdisciplinary Graduate School (IGS), Nanyang Technological University (NTU), Singapore, Singapore; 2 Singapore Centre for Environmental Life Sciences Engineering (SCELSE), Nanyang Technological University (NTU), Singapore, Singapore; 3 St John’s Island National Marine Laboratory (SJIMML), Tropical Marine Science Institute (TMSI), National University of Singapore (NUS), Singapore, Singapore; 4 Centre for Life Sciences (CELS), National University of Singapore (NUS), Singapore, Singapore; 5 Singapore Centre for Environmental Life Sciences Engineering (SCELSE), National University of Singapore (NUS), Singapore, Singapore; 6 School of Biological Sciences (SBS), Nanyang Technological University (NTU), Singapore, Singapore; 7 Department of Biochemistry, National University of Singapore (NUS), Singapore, Singapore

**Keywords:** Next-generation sequencing, Metagenomics, Water microbiology

## Abstract

Methods for the study of member species in complex microbial communities remain a high priority, particularly for rare and/or novel member species that might play an important ecological role. Specifically, methods that link genomic information of member species with its spatial structure are lacking. This study adopts an integrative workflow that permits the characterisation of previously unclassified bacterial taxa from microbiomes through: (1) imaging of the spatial structure; (2) taxonomic classification and (3) genome recovery. Our study attempts to bridge the gaps between metagenomics/metatranscriptomics and high-resolution biomass imaging methods by developing new fluorescence in situ hybridisation (FISH) probes—termed as R-Probes—from shotgun reads that harbour hypervariable regions of the 16S rRNA gene. The sample-centric design of R-Probes means that probes can directly hybridise to OTUs as detected in shotgun sequencing surveys. The primer-free probe design captures larger microbial diversity as compared to canonical probes. R-Probes were designed from deep-sequenced RNA-Seq datasets for both FISH imaging and FISH–Fluorescence activated cell sorting (FISH–FACS). FISH–FACS was used for target enrichment of previously unclassified bacterial taxa prior to downstream multiple displacement amplification (MDA), genomic sequencing and genome recovery. After validation of the workflow on an axenic isolate of *Thauera* species, the techniques were applied to investigate two previously uncharacterised taxa from a tropical full-scale activated sludge community. In some instances, probe design on the hypervariable region allowed differentiation to the species level. Collectively, the workflow can be readily applied to microbiomes for which shotgun nucleic acid survey data is available.

## Introduction

The study of complex microbial communities typically involves a combination of analyses needed to gain a comprehensive understanding of their composition, structure and function.^1^ These analyses include: (1) taxonomic identification of member species and the ability to monitor their changes in abundance temporally and spatially^2^; (2) genome recovery of member species, including the identification of genes and their coding sequences^3,4^ and (3) microscopic imaging of member species to understand their spatial organisation and structural interrelationships with members from other taxa.^5^

Taxonomic identification of microbes has largely relied on the phylogenetic analysis of 16S rRNA gene sequences,^6^ which provides a template for the design of fluorescence in situ hybridisation (FISH) probes. FISH is commonly used to visualise the three-dimensional spatial distribution of targeted microbes within complex communities with microscopy.^7–9^ However, 16S rRNA gene sequences obtained from amplicon sequencing of specific hypervariable regions,^10^ or even recent advanced full-length reconstruction techniques^11^ do not provide insight into the functional potential of the community.

While shotgun metagenomics permits the genome recovery of member species, it is difficult to resolve the genome organisation of communities with high diversity and high evenness.^4^ Quite often, the genomes of rare OTUs,^12^ that often represent novel taxonomic entities^13^ in the long tail of rank-abundance plots of community analyses cannot be effectively recovered; only near-complete genomes belonging to the dominant members can be recovered through genomic binning. In summary, it is challenging to combine current approaches to perform taxonomic assignment, genome recovery and imaging of rare member species in complex communities.

Given the large-scale deployment of high-throughput shotgun sequencing for microbial community profiling, the resulting short length reads derived from hypervariable regions of the SSU gene present an opportunity for the design of FISH probes, as demonstrated by Hasegawa et al.^14^ using 16S rRNA gene amplicon sequences. In this study, FISH probes are designed directly from short sequence reads obtained from shotgun sequencing dataset (metagenomics or metatranscriptomics) without the potential complications introduced by amplicon sequencing. This strategy is sample specific and deviates from the conventional primer-based approach of designing FISH probe from comparative sequence analysis with a global reference database.^15^ Nomenclature of probes designed through this study are designated as R-Probes. Being a sample-centric approach, R-Probes can target OTUs detected directly from shotgun sequencing surveys. R-Probes were designed from taxonomically informative tag sequences (33 bp) from the hypervariable region (V4–V7) of the 16S rRNA as defined by our previously developed RiboTagger software.^16^ The RiboTagger software was selected because of its faster processing time compared to other similar software, and the use of universal recognition profile with a high sensitivity for the extraction of tag sequences. Furthermore, if samples have been sequenced to a high depth, probes can be designed against rare members of the communities.

Following validation studies using axenic cultures, the method was applied to study two previously uncharacterised microbial taxa (not represented in the SILVA SSU^17^ and the NCBI Genome database^18^) from an activated sludge community sampled from a full-scale wastewater plant. The activated sludge community was selected because an ultra-deep metagenomic–metatranscriptomics sequencing revealed a large degree of taxonomic novelty. Spatial structures of the target microbial groups were studied using R-Probes, and FISH–Fluorescence activated cell sorting (FISH–FACS) was used to obtain the “mini-metagenome” prior to downstream multiple displacement amplification (MDA), genomic sequencing and analyses.

## Results

### Design of R-Probes

The highly diverse, floccular microbial community sampled from a full-scale wastewater treatment plant in Singapore (see Methods) was used as a proof of concept environmental sample for R-Probe design. Initial 16S rRNA sequence profiling was performed with the RiboTagger software^16^ to examine the diversity in these samples, using adaptor- and quality-trimmed metatranscriptomics reads obtained from multiple RNA-seq datasets of the microbial community. The RiboTagger software detects sequence tags from the V4–V7 variable regions of the 16S rRNA (termed ribotags). In this study, the V6 hypervariable region was used. Each identified sequence tag has a default length of 33 bp, and in most instances, an OTU is defined by its unique 33bp ribotag sequence.

R-Probes were subsequently designed from ribotags, where the tag sequence served as template for FISH probe design. In this study, the 33 bp ribotag was truncated to fit into the melting curve profiles of other published FISH probes: EUB338 or Thau646 (typical read length of 17–25 bp) for co-hybridisation studies and probe evaluation. This was performed to attain stringent hybridisation at a standardised hybridisation temperature of 46 °C.^19^ Specifically, R-Probes of 17 bp were designed by truncating the 3′ end of the 33 bp ribotag (Supplementary Fig. 1). Truncating the length of 33bp ribotag sequence might lead to the hybridisation of other OTUs that share the same R-Probe sequence, thus affecting the original specificity of the FISH probe and subsequent genomic analyses (see next section in Results and Supplementary Fig. 2 for influence of probe truncation).

R-Probes were applied to the following microbial groups: (1) *Thauera* OTU; (2) an unclassified bacterial taxon and (3) a novel OTU of the genus *Haliangium* (Table 1). The TestProbe tool^17^ was used to check the in silico coverage and specificity of probes, with the SILVA 123 SSU Ref NR99 database as a reference. Nomenclature of R-Probes is as follows:

**Table 1:** FISH probes and ribotags used

FISH probes/ribotags	Intended target taxon	Sequence of probe (5′→3′)	Formamide (%)	References
EUB338	Most bacteria	GCTGCCTCCCGTAGGAGT	0	Amann et al.^7^
NON338	None	ACTCCTACGGGAGGCAGC	45	Wallner et al.^8^
Thau646	*Thauera*	TCTGCCGTACTCTAGCCTT	45	Lajoie et al.^43^
Ribo_Thau1029_33^a^	*Thauera*	GTGTTCTGGCTCCCGAAGGCACCCTCGCCTCTC	N.D	This study
Ribo_Thau1029_17	*Thauera*	GTGTTCTGGCTCCCGAA	45	This study
Ribo_Unk1029_33^a^	UPWRP_1	TGCTTCGCGTCTCCGAAGAGCCGACCACCTTTC	N.D	This study
Ribo_Unk1029_17	UPWRP_1	TGCTTCGCGTCTCCGAA	50	This study
Halian2^b^	*Haliangium*	CCGACTTCTAGAGCAACTGA	25	McIlroy et al.^41^
Halian3^b^	*Haliangium*	CCAGTCACTCTTTAGGCGGC	25	McIlroy et al.^41^
Ribo_Halia1029_33^a^	UPWRP_2	TCTCACTCGCTCCCGAAGGCACCCCGACATCTC	N.D	This study
Ribo_Halia1029_17	UPWRP_2	TCTCACTCGCTCCCGAA	40	This study
Halia183	UPWRP_2	GAAATCCGGAAACCTCACAGAC	40^c^	This study

*N.D* not determined

^a^Original 33 bp ribotag of the respective R-Probe

^b^Probes are components of the HalianMix

^c^Optimal formamide concentration was not empirically determined

1. “Ribo” to be added in front of the probe name, followed by an underscore

2. Probe name as described by Alm et al.,^20^ followed by an underscore

3. Length of the probe

All FISH probes were synthesised as DNA oligonucleotides and purified by high-pressure liquid chromatography (Integrated DNA Technologies, USA). Oligonucleotide sequences and optimal hybridisation conditions of FISH probes used are listed in Table 1.

### Validation of R-Probe and FISH–FACS optimisation using a reference taxonomic group

**Fig. 1:**
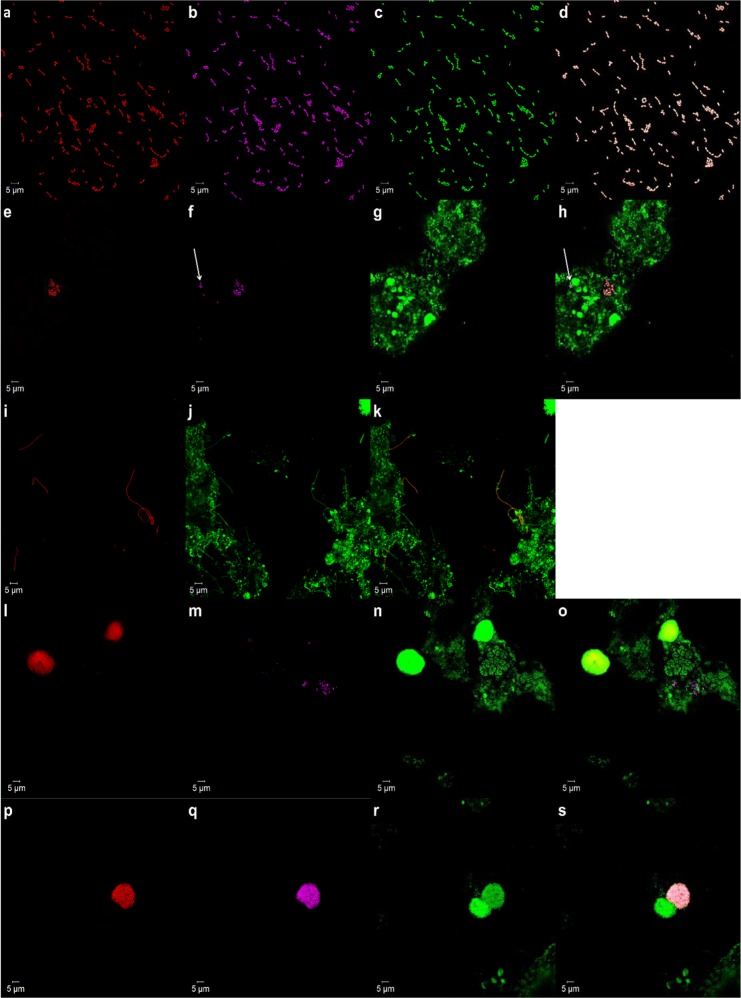
Confocal micrographs depicting the hybridisation of R-Probes and published FISH probes to paraformaldehyde-fixed samples. **a**–**d** An axenic culture of *Thauera* sp. R086 hybridised with probes Ribo_Thau1029_17_Cy5_ (red), Thau646_Cy3_ (magenta) and EUB338_A488_ (green). **e**–**h** *Thauera* sp. in an activated sludge sample, hybridised with probes Ribo_Thau1029_17_Cy5_ (red), Thau646_Cy3_ (magenta) and EUB338_A488_ (green). Arrow-pointed cells were hybridised with Thau646_Cy3_, but not with Ribo_Thau1029_17_Cy5_. **i**–**k** UPWRP_1 in an activated sludge sample, hybridised with probes Ribo_Unk1029_17_Cy5_ (red) and EUB338_A488_ (green). **l**–**o** *Haliangium* sp. in an activated sludge sample, hybridised with probes Ribo_Halia1029_17_Cy5_ (red), HalianMix_Cy3_ (magenta) and EUB338_A488_ (green). **p**–**s** Interaction of UPWRP_2 with a cell cluster in an activated sludge sample, hybridised with probes Ribo_Halia1029_17_Cy5_ (red), Halia183_Cy3_ (magenta) and EUB338_A488_ (green). Images (**d**), (**h**), (**k**), (**o**), (**s**) represent superimposition of their respective image set. All figures have a scale bar representing 5 µm, and images were taken with ×630 magnification

*Thauera* sp. was selected as a reference taxonomic group for method optimisation as it was ranked as a dominant member of the floccular sludge community. R-Probe, Ribo_Thau1029_17_Cy5_ was designed from the most abundant RiboTagger-obtained V6 sequence annotated to the genus *Thauera* (see Methods). Ribo_Thau1029_17_Cy5_ was applied to an axenic culture of *Thauera* sp. R086, which was co-hybridised with published probes, Thau646_Cy3_ and EUB338_A488_. Confocal images showed Ribo_Thau1029_17_Cy5_-labelled cells being overlapped with fluorescent signals from the published probes (Fig. 1a–d), thus providing evidence that the R-Probe was targeting the genus *Thauera*. Specificity of the R-Probe was evaluated with a non-target organism, *Thauera linaloolentis*, which had two bp mismatches with Ribo_Thau1029_17_Cy5_ (Supplementary Table 1)_._ No fluorescence signals were detected in the negative control using the same microscopic settings (Supplementary Fig. 3).

Confocal imaging with the same set of probes was applied to an activated sludge sample, and Ribo_Thau1029_17_Cy5_ overlapped with probe Thau646_Cy3_ in most EUB338-labelled cells (Fig. 1e**–**h). Quantitative co-localisation analysis of microscopic images revealed that majority of R-Probe-labelled cells (98.81 ± 0.037%, *n* = 66, mean ± SD) co-localised with a smaller fraction of Thau646-labelled cells (80.47 ± 0.013%, *n* = 66, mean ± SD) (Supplementary Fig. 4). The co-localisation results presented two scenarios: potential problem of non-specificity binding of Thau646_Cy3_ in a mixed microbial community setting or inadequate coverage of *Thauera* sp. by Ribo_Thau1029_17_Cy5_.

The FISH–FACS enrichment process was optimised on activated sludge samples to enrich for *Thauera* sp. Gate-guided sorting resulted in a clear and distinct population appearing with enhanced signal on the Cy5 and A488 axis after hybridisation of probes Ribo_Thau1029_17_Cy5_ and EUB338_A488_ (Supplementary Fig. 5). Microscopic visualisation of sorted cells confirmed the presence of positively labelled single cocci cells, with co-localisation in both the Cy5 and Alexa 488 filter set (Supplementary Fig. 6a**–**c).

**Fig. 2:**
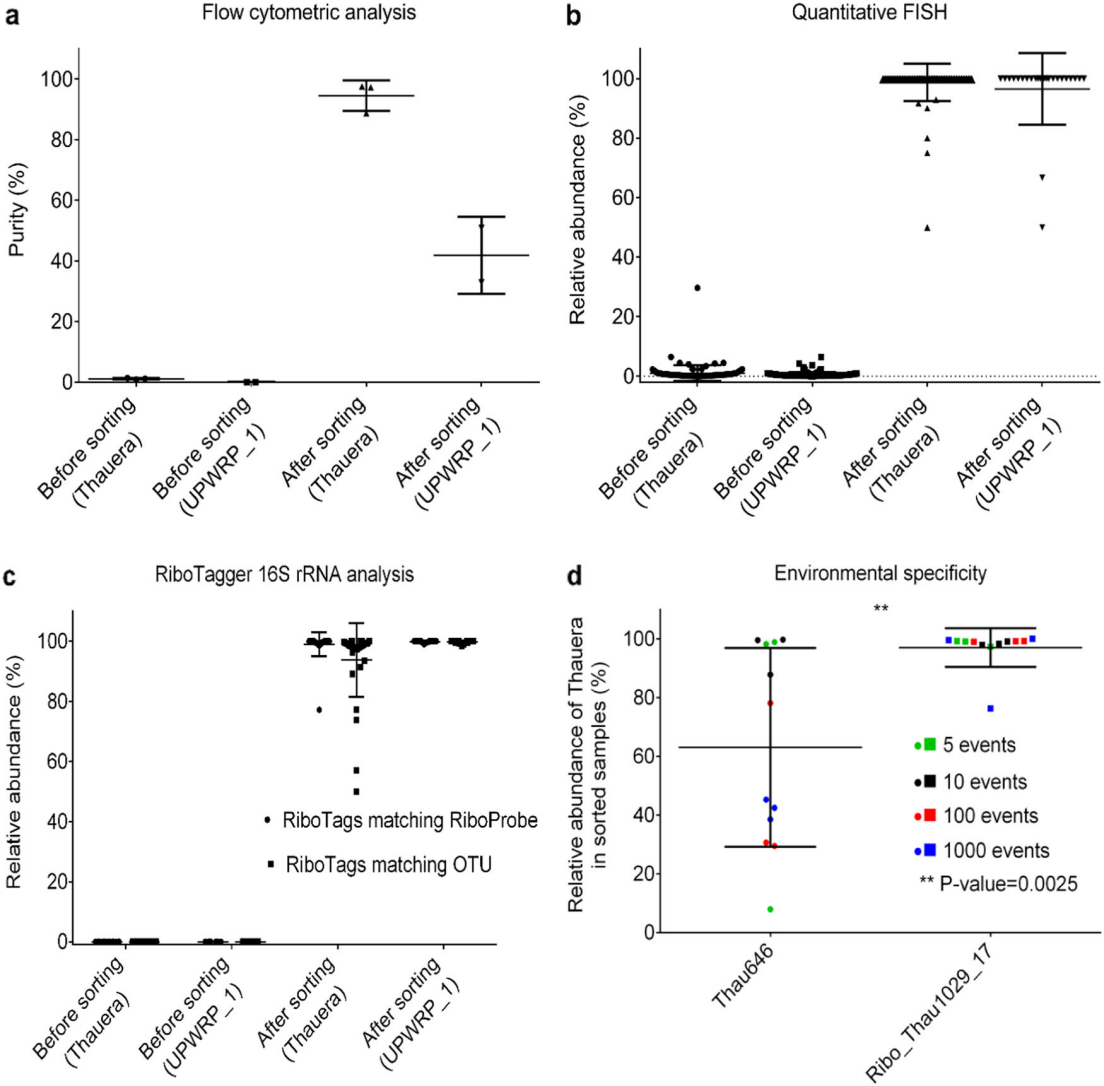
Effectiveness of cell sorting and enrichment level of cells that have been processed with FISH–FACS. **a** Cell sorting purity obtained after an initial round of sorting was calculated from flow cytometric analysis. **b** Quantitative FISH analysis depicting the relative abundance of target cells in pre-sorted and sorted samples. Quantitative FISH analysis was performed by acquiring confocal images, followed by biovolume image analysis. Each dot represents quantitative FISH analysis performed on one confocal microscope image. **c** Relative abundance of ribotags matching the R-Probe and OTU in metagenomic DNA of pre- and post-sorted samples. Each dot represents a sequenced sample. **d** Comparison of the environmental specificity of probes Thau646 and Ribo_Thau1029_17. The sorting was performed in various event cut-offs and the legend denotes the different number of sorted events

The effectiveness of FISH–FACS sorting was evaluated as follows: (1) sorting purity obtained from flow cytometric analysis; (2) quantitative FISH analysis of pre- and post-sorted samples and (3) 16S rRNA profiling of genomic DNA obtained from pre- and post-sorted samples (Fig. 2a**–**c). Sorting purity is defined as the percentage of events that mapped back to the sorting gates that were defined during the initial FACS sorting. The purity assessment indicated that probe-labelled cells were enriched from an initial abundance of 1.15% ± 0.24 to 94.53% ± 5.05 (*n* = 3, mean ± SD) after an initial round of sorting (Fig. 2a). This result corroborates with quantitative FISH analysis where *Thauera* cells were enriched up to 93 fold, from a relative abundance of 1.06% ± 0.23 (*n* = 135, mean ± SEM) in pre-sorted samples to 98.66% ± 0.67 (*n* = 90, mean ± SEM) in sorted samples (Fig. 2b).

Metagenomic sequencing was performed on pre-sorted and sorted samples, and subsequently analysed with 16S rRNA profiling. Five OTUs (*n* = 10 V6 sequence tags) were detected in the pre-sorted samples, and none belonged to *Thauera* sp. The small number of sequence tags could be attributed to the highly complex community composition and low-sequencing depth used (Supplementary Table 2). Sorted samples that were MDA amplified had a total of 66 unique OTUs identified (Supplementary Table 3). Three OTUs were classified to the genus *Thauera* and one OTU to the genus *Leptolyngbya*. The other OTU was classified to the family level: Rhodocyclaceae. The remaining OTUs lacked taxonomical affiliations at all levels. In summary, FISH–FACS sorting with Ribo_Thau1029_17 yielded high specificity for the probe targeted taxon, as evidenced by having more than 93% of total V6 sequence tags being matched to Ribo_Thau1029_17 (98.97% ± 4.00) and its target 33 bp ribotag *Thauera* OTU (93.79% ± 12.20, *n* = 32, mean ± SD) (Fig. 2c). The discrepancy between the two figures indicates that the other OTUs that shared Ribo_Thau1029_17 sequence were co-sorted with the target *Thauera* OTU.

Whole genome sequencing of MDA-amplified DNA extracted from Ribo_Thau1029_17-sorted samples (*n* = 27) produced 2223 contigs with an N50 of 5692 bp (Supplementary Table 4). Subsequent tetra-nucleotide frequency (TNF)-binning produced three genomic bins, where two of the bins were taxonomically affiliated with *Thauera*. One of the genomic bins affiliated with *Thauera* had a genome completeness of 38.84%, contamination of 2.35% and a GC content of 67.59% (Table 2).

**Table 2:** Statistics of the draft genomes of *Thauera*, UPWRP_1 and UPWRP_2

	Draft genomes		
	*Thauera*	UPWRP_1	UPWRP_2
Number of contigs	349	424	462
Genome size (Mbp)	2.24	10.16	2.37
GC content (%)	67.59	45.85	63.72
N50 of contigs (bp)	8,170	37,539	12,548
Largest contig (bp)	30,474	145,517	55,295
Number of essential single-copy genes	48/111	204/111	41/111
Number of unique single-copy genes	42	104	41
Genome completeness (%)	38.84	98.28	21.70
Genome contamination (%)	2.35	94.48	0.02
Number of ORFs	2,073	7,535	2,081
Number of tRNA	32	79	7
Number of rRNA	5	2	3
Phylogenetic placement in reference genome tree	k_Bacteria; p_Proteobacteria; c_Betaproteobacteria; o_Rhodocyclales; f_Rhodocyclaceae; g_*Thauera*; s_*Thauera aminoaromatica* (sister lineage)	k__Bacteria; p__Bacteroidetes; c__Sphingobacteriia; o__Sphingobacteriales; f__Saprospiraceae (sister lineage)	k_Bacteria; p_Proteobacteria; c_Deltaproteobacteria; o_Myxococcales; f_Kofleriaceae; g_Haliangium

### Specificity comparison between R-Probe and published probe for *Thauera* sp

The specificity of Ribo_Thau1029_17 and Thau646 for *Thauera* sp. in activated sludge samples was further evaluated. RiboTagger 16S rRNA analysis indicated that only 63.01% ± 33.82 (*n* = 12, mean ± SD) of V6 sequence tags from Thau646-sorted samples were classified to *Thauera* (Fig. 2d). A statistically significant difference (*p* = 0.0025) was found between the two groups of sorted samples. The most abundant non-target taxon co-sorted with Thau646 was identified to be a phylogenetically distant *Aquabacterium* sp. (15.23%). Comparative sequence analysis revealed that Thau646 binding site is present in the V4 region of the 16S rRNA of *Aquabacterium*. RiboTagger results corroborates with qualitative co-localisation microscopic analysis (Supplementary Fig. 4). In conclusion, Ribo_Thau1029_17 had a significantly higher specificity than Thau646 for *Thauera* sp. in activated sludge samples.

### Visualisation of novel bacterial OTU using R-Probes

The established procedures developed above were subsequently employed to visualise and enrich for two novel OTUs identified in activated sludge samples. The OTUs were selected as examples to demonstrate the feasibility of using R-Probes to target and characterise novel OTUs. An unannotated V6 sequence, denoted as UPWRP_1 that was present at 0.77% relative abundance in the metatranscriptomic dataset was used as a template for the design of probe Ribo_Unk1029_17. Confocal images revealed Ribo_Unk1029_17_Cy5_-labelled cells displaying a filamentous morphology. The filaments observed were mostly single filaments, and with some existing as aggregates (Fig. 1i–k). Quantitative FISH indicated that UPWRP_1 constituted between 0.60 ± 0.070% (*n* = 140, mean ± SEM) of the total bacterial biomass prior to enrichment (Fig. 2b), which is consistent with its relative abundance in the metatranscriptomics dataset.

### Unusual aggregated structure of an uncharacterised *Haliangium* sp. from an activated sludge community of a full-scale wastewater treatment plant

The same approach was used to study a member of the genus *Haliangium* present in the community (UPWRP_2, 2.35% relative abundance in the metatranscriptomics dataset) through the design of a probe denoted as Ribo_Halia1029_17. Confocal images revealed Ribo_Halia1029_17-labelled cells formed dense cell clusters, with individual *Haliangium* cells being rarely observed (Fig. 1l–o). Co-hybridisation with published *Haliangium* probes (HalianMix) revealed (*n* = 12, mean ± SD) different spatial appearances of HalianMix-labelled cells (predominantly single cells or small clusters) and no signal overlap between Ribo_Halia1029_17 and HalianMix-labelled cells (Fig. 1l–o). Cells labelled with both probes were spatially distinct, therefore implying that the probes targeted different clades within the genus. This was verified though probe sequence matching of 16S rRNA gene sequences of *Haliangium* sp. in the ARB-parsimony tree (Supplementary Fig. 7). Different morphologies of *Haliangium* cells: spherical-, rod- and coccus-shaped could be observed through microscopy (Supplementary Fig. 8) and differentiated using flow cytometer (Supplementary Fig. 9).

In addition to UPWRP_2, other *Haliangium* sp. were also targeted by Ribo_Halia1029_17 (Supplementary Table 5). This was also evidenced through co-sorting of another *Haliangium* species (accession number: AB286567) through FISH–FACS (Supplementary Table 6). Subsequently, a specific probe, Halia183 (Table 1) was designed to target UPWRP_2. Through co-hybridisation of probes Halia183_Cy3_, Ribo_Halia1029_17_Cy5_ and EUB338_A488_, UPWRP_2 was shown not to co-exist in a cell cluster with other *Haliangium* sp. in all confocal images (Fig. 1p–s). However, clusters of UPWRP_2 formed interactions with other bacterial cell clumps. To date, the taxa of the interacting cell clusters have not been identified.

### Enrichment of a novel bacterial taxon (UPWRP_1) from an activated sludge community of a full-scale wastewater treatment plant

**Fig. 3:**
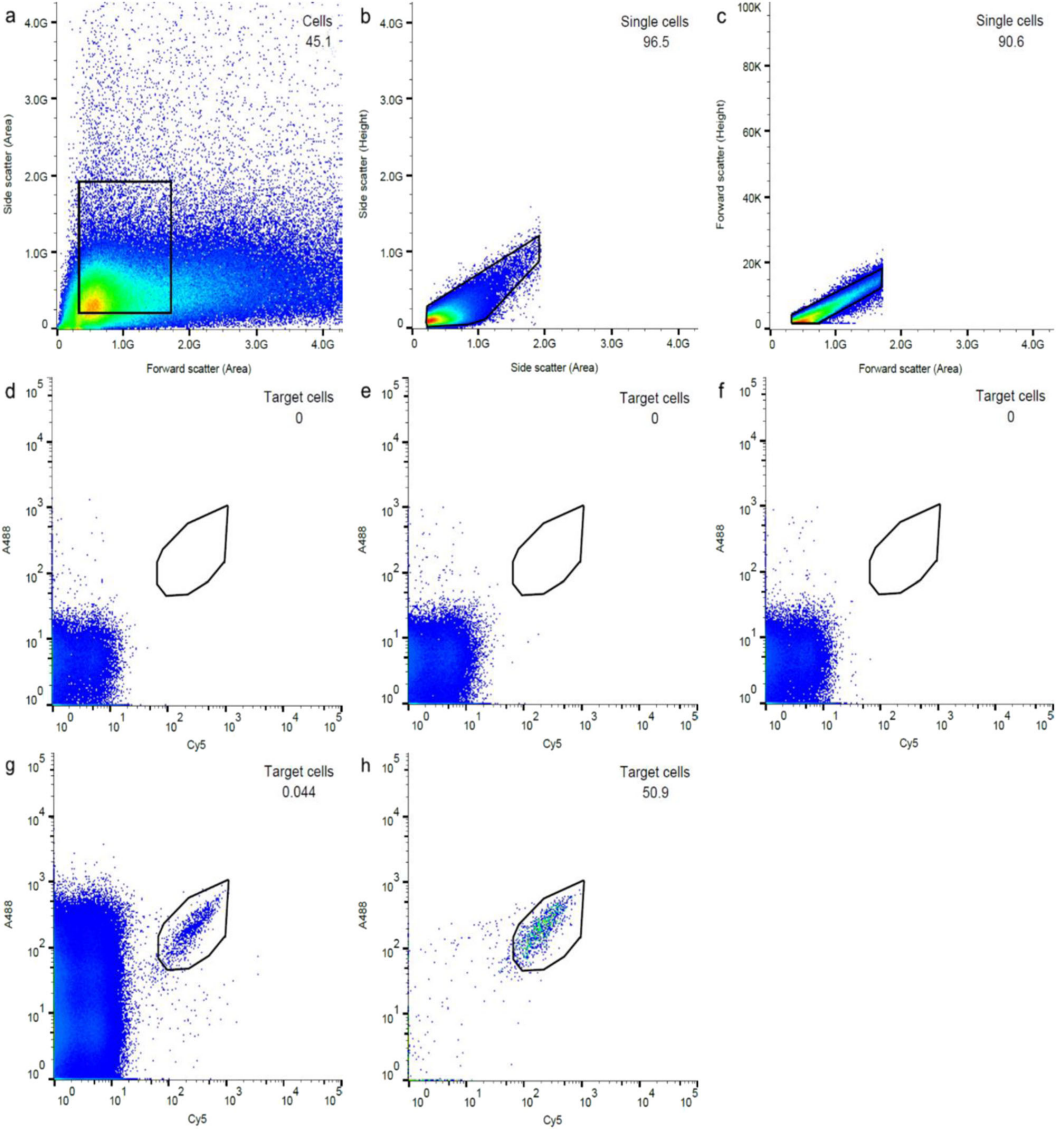
Flow cytometric analysis of sorting UPWRP_1 from an activated sludge sample hybridised with probes Ribo_Unk1029_17_Cy5_ and EUB388_A488_. An ~100,000 events were collected for each FACS plot, except for the purity check. Sorting gates are outlined in black and values shown indicate the percentage of gated events over the total number of events. Flow cytometric analysis: **a** Sorting gate 1: sorting of bacterial cells using their light-scattering properties with a plot of forward versus side scatter; **b** sorting gate 2: filtering out cell aggregates based on side scatter area versus side scatter height; **c** sorting gate 3: filtering out cell aggregates based on forward scatter area versus forward scatter height. Sorting gate 4 was constructed to exclude events exhibiting Cy5 and A488 fluorescence signal in the negative controls: **d** no-probe control; **e** hybridisation with probe NON338_Cy5_ to estimate non-specific binding for Cy5 fluorophore; **f** hybridisation with probe NON338_A488_ to estimate non-specific binding for A488 fluorophore. **g** Events exhibiting Cy5 and A488 fluorescence signal above the cut-off threshold for the negative controls were collected in sorting gate 4. **h** Purity of the sorted sample after an initial round of sorting

FISH–FACS procedures were applied to enrich for UPWRP_1 using Ribo_Unk1029_17, with an additional pre-treatment procedure (see Methods) to disperse the aggregated filaments (Supplementary Fig. 6d–f) in order to facilitate the sorting process. Sorted cells retained their filamentous morphology after the sorting process (Supplementary Fig. 6g–i). A higher level of background noise was observed in the forward scatter during sorting (Fig. 3), and this was probably due to an additional pre-treatment procedure. Consequently, the threshold of the forward scatter had to be increased, resulting in a lower sorting purity for the initial round of sorting (Figs 2a and 3g–h).

Nevertheless, both quantitative FISH and RiboTagger 16S rRNA analyses indicated high enrichment of the targeted taxon in samples that were subjected to two rounds of sorting (Fig. 2b, c). Relative abundance of V6 sequence tags matching to Ribo_Unk1029_17 (99.87% ± 0.28) and UPWRP_1 (99.67% ± 0.48, *n* = 32, mean ± SD) were similar, thus indicating that Ribo_Unk1029_17 was highly specific for UPWRP_1. Similar to the *Thauera* enrichment, pre-sorted samples contained no sequence tags matching to Ribo_Unk1029_17. This is probably due to the low abundance of UPWRP_1 and high complexity of the activated sludge community (Supplementary Table 2). Samples sorted with probe Ribo_Unk1029_17 produced 15 OTUs (Supplementary Table 7).

### 16S rRNA phylogenetic analysis of UPWRP_1-enriched samples

**Fig. 4:**
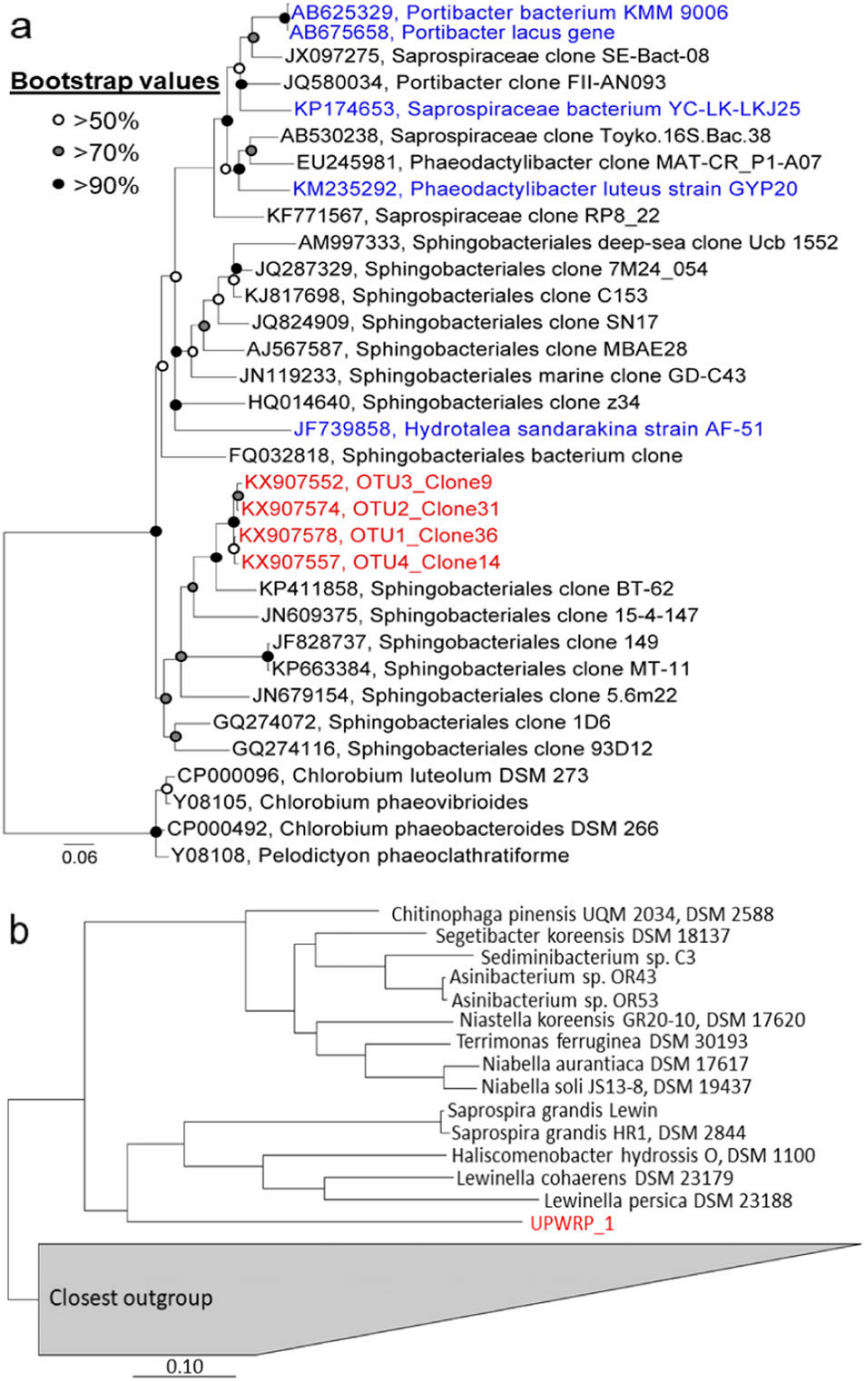
Phylogenetic analysis of marker genes retrieved from UPWRP_1. **a** Maximum-likelihood (PhyML) phylogenetic tree depicting the 16S rRNA gene phylogenetic relationship of UPWRP_1 and its closely related sequences in the SILVA 123 SSU Ref NR99 database. Members of the genus *Chlorobium* were used as the outgroup. Only near full-length sequences (≥1200 bp) were selected. Representative sequences of OTUs are demarcated in red, and sequences of the closest cultured isolates are demarcated in blue. Bootstrap values were calculated from 1000 bootstrap analyses, and only bootstrap values over 50% were displayed. Branches with low bootstrap values ≤50% have been multifurcated. The scale bar represents substitutions per nucleotide base. Legend of the bootstrap values is located at the upper left-hand corner of the diagram. **b** Phylogenetic placement of genomics bin of UPWRP_1 in the reference genome tree using a concatenation of 43 phylogenetic marker genes. A set of 2502 complete genomes and 3604 draft genomes from IMG database were used in the reference genome tree. Genomic bin of UPWRP_1 is demarcated in red. Closest outgroup corresponded to different classes under the phylum Bacteroidetes

To taxonomically identify UPWRP_1, a 16S rRNA clone library was constructed and de novo OTU clustering of sequenced clones at 99% sequence similarity resulted in four representative OTUs. The four OTUs were subdivided into two clades, and the 16S rRNA gene sequences have sequence similarity ranging from 98.11 to 98.97% (Supplementary Table 8). The OTUs formed an independent monophyletic clade that clustered separately from its closest relative, Sphingobacteriales clone BT-62 (accession number: KP411858), which shared sequence similarity of 90.63–91.11% (Fig. 4a). The closest cultured isolates to the four OTUs is *Portibacter lacus* (accession number: AB675658), with a sequence similarity of 82.12–82.52%. The OTUs are affiliated to the order Sphingobacteriales, class Sphingobacteriia and of the phylum Bacteroidetes. Taxonomical information at the family or genus level could not be inferred.

### Draft genome analysis of UPWRP_1

A metagenomic co-assembly of Ribo_Unk1029_17-enriched samples produced 1302 contigs with a total length of 12.7 Mbp, an N50 contig size of 32,430 bp and the longest contig with a length of 145,517 bp (Supplementary Table 4). Using TNF-only binning, a single genomic bin with a completeness of 98.28% and a contamination of 94.48% was recovered. The high contamination level contrasted the results of RiboTagger 16S rRNA analysis, which showed that the sorted samples were highly enriched for UPWRP_1 as majority of the ribotags (99.56% ± 0.82, *n* = 16, mean ± SD) were classified to UPWPR_1.

An analysis with CheckM revealed that 99 out of 104 lineage-specific marker genes were repeated twice, thus raising the possibilities of two different genomes. The total number of essential single-copy genes (*n* = 204) also suggested the presence of two genomes (Table 2). Multicopy genes were aligned, and a frequency distribution plot of the average amino acid identity (AAI) is presented in Supplementary Fig. 10a. The mean, median, 25th percentile and 75th percentile of the AAI were above 0.94 (Supplementary Fig. 10b), therefore suggesting that the two genomes present were closely related to each other at the species level.^21^ As the genomic sequences were amplified with MDA, differential coverage binning was not attempted as it is likely that multiple, incomplete genome bins would be generated due to uneven coverage distribution.^22^ The sequencing coverage of the draft genome of UPWRP_1 increased from 0.57 ± 6.13 in pre-sorted samples to 525.85 ± 594.72 in sorted samples, therefore supporting the enrichment process of UPWRP_1 (Supplementary Table 2).

Analysis of a concatenated set of phylogenetic conserved marker genes resulted in the phylogenetic placement of UPWRP_1 to the order Sphingobacteriales, of the class Sphingobacteriia and of the phylum Bacteroidetes (Table 2). The closest genome to UPWRP_1 is *Lewinella persica* DSM 23188 (IMG 2515154070) from the sister lineage genus *Lewinella*, under the family *Saprospiraceae* (Fig. 4b). UPWRP_1 and *Lewinella persica* DSM 23188 shared a 16S rRNA gene sequence similarity of 81.95%. Due to the deep branch placement of UPWRP_1 in the phylogenetic tree, the genomic bins can be categorised as a novel genus and family.

### Enrichment and genome recovery of the uncharacterised *Haliangium* sp

Similar FISH–FACS procedures were applied to enrich for UPWRP_2 using probe Ribo_Halia1029_17. RiboTagger 16S rRNA analysis showed that only 44.14% of V6 sequence tags could be annotated to UPWRP_2 (Supplementary Table 6). Lower-than-expected level of enrichment for UPWRP_2 is consistent with co-sorting of another taxon (relative abundance: 44.86%; accession number: AB286567) which possessed the binding site for probe Ribo_Halia1029_17. Both UPWRP_2 and *Haliangium* species (accession number: AB286567) shared a 16S rRNA gene sequence similarity of 93.11%. Approximately 11% of the ribotags could not be annotated. A total of 56 out of the 70 non-annotated ribotags have long representative sequences that were closely related (>98% similarity) with UPWRP_2. This was achieved through multiple sequence analyses of the long representative sequence of UPWRP_2 and the non-annotated ribotags. However, it should be noted that pairwise analyses were performed with the representative sequence of a read length of only 81 bp long, and is therefore not very informative without the full-length 16S rRNA sequences.

A clone library was generated from a sorted sample, where 100% of V6 sequence tags matched UPWRP_2. De novo OTU clustering resulted in a single OTU (accession number: KX954253). The OTU formed a monophyletic clade with its closest neighbour: *Haliangium* clone 0102 (accession number: AB286332) with a sequence similarity of 98.37%, and the phylogenetic placement was supported by a high bootstrap value of >90% (Supplementary Fig. 11). Due to lower-than-expected level of enrichment for UPWRP_2 (Supplementary Table 6), co-assembly of multiple sorted samples was not attempted. The same sample used for 16S rRNA gene phylogenetic analysis was selected to generate a draft genome of UPWRP_2. The total number of essential single-copy genes was equivalent to the number of unique essential single-copy genes (*n* = 41), thus indicating the presence of only one genome (Table 2). This was further validated with CheckM which showed a genome completeness of 21.70% and a contamination of only 0.02%.

## Discussion

In this study, existing gaps between whole community shotgun sequencing and high-resolution biomass imaging methods were bridged by taking advantage of FISH probes designed directly from 16S rRNA gene variable regions obtained from shotgun sequencing data. Newly designed probes were applied to explicitly target specific taxa in the community for microscopic imaging and FISH–FACS for genome recovery. This permitted the characterisation of two previously unclassified microbes: one from the order Sphingobacteriales (UPWRP_1) and the other from the genus *Haliangium* (UPWRP_2).

While FISH probes have previously been designed from the V6 region of 16S rRNA gene from pyrosequences,^14^ the motivation for the design of R-Probes is to mitigate bias that may be introduced during PCR amplification of the hypervariable region through the extraction of taxonomically informative tag sequences directly from shotgun sequencing data.^16^ Furthermore, the FISH probes designed by Hasegawa et al.^14^ target only the abundant organisms. R-Probes can be complemented with the high-sequencing depth obtained with metagenomics/metatranscriptomics to target taxonomic novel entities present at low abundance. Furthermore, the design of R-Probes from ribotags is performed with minimal data processing and ribotags can be used directly as templates for FISH probe design.

Another advantage of R-Probes lies in its sample-centric design as compared to conventional probe design which relies on a public database. The number of potential non-target taxa increases with use of a larger database, thus making probe design for an intended target group a challenge. The use of a curated sample-centric database would benefit FISH probe design as more accurate information pertaining to the in silico specificity and coverage can be assigned to FISH probes.

R-Probes also offer the advantage of visualising the target OTU to confirm its presence as detected in shotgun sequencing surveys. V6-R-Probes conferred bright fluorescence signal to three phylogenetically distant bacterial taxa (*Thauera, Haliangum*, and UPWRP_1) in FISH microscope images. This visual evidence demonstrates that R-Probes could be used to target members using specific unique tag sequences from the hypervariable regions of the 16S rRNA gene. The V6 region was selected among the V4–V7 regions because it was better suited to estimating taxa richness at the species level,^6^ and the 16S secondary structure model in this region had demonstrated moderate accessibility of FISH probes.^23^ Furthermore, the V6 region provides a meaningful taxonomic resolution that can distinguish between closely related species, as shown in the case study of UPWRP_2 which shared a 16S rRNA sequence similarity of 98.37% to its closest neighbour.

Obtaining draft genomes of previously unidentified taxa is important in microbial community analysis because it provides a reference for phylogenetic assignment of genomic fragments for future metagenomics surveys. The recovery of high-quality draft genomes of member species of microbial communities from shotgun sequencing data^24^ is limited by: (1) the sequencing depth required to access the genomes of targeted members of the community and (2) the difficulties of recovering member genomes from metagenomes in microbial communities with moderate-to-high eco-genetic complexity.^3,25^

In most settings that do not involve community simplification (e.g. enrichment reactors^26^), only near-complete genomes belonging to the dominant taxa can be recovered. Targeted genomics approaches^27,28^ such as FISH–FACS or single-cell genomics methods^13^ can reduce the genome complexity of the community and enrich for rare taxa. Our study differs from other FISH–FACS studies^29–32^ because of our approach to designing new FISH probes from high-throughput sequencing dataset for downstream genome recovery of previously uncharacterised taxa. In contrast, most FISH–FACS studies use existing broad-based taxa FISH probes to hybridise to target organisms that have established taxonomic identities. Due to the evolutionary conservation of the 16S rRNA molecule, genomic strain diversity cannot be fully dissected with FISH probes.

Complementary to the use of FISH–FACS for genome recovery, single-cell genomics (SCG) avoids the complications of genetic heterogeneity for downstream genome analysis,^33^ and overcomes the limitations of FISH such as high background fluorescence and low fluorescence of target cells.^34^ However, an important trade-off is the additional technical complexity and resources required for cell isolation, followed by functional or phylogenetic-based screening of single cells. Although single-cell sorting could be applied with FISH for taxonomy-based fluorescent labelling,^35^ we demonstrated low consistency of successful genome amplification.

Genomic divergence in the sorted samples would complicate the genome assembly of repetitive regions in closely related strains,^36^ and result in a low genome completeness as shown with the genomic bins of *Thauera*. Strain heterogeneity was also present in the sorted samples of UPWRP_2, where majority of the non-annotated ribotags had a close sequence similarity to UWRP2. Multiple sequence alignment of the long representative sequences of UPWRP_2 and selected non-annotated ribotags showed that 56 out of 70 non-annotated ribotags yielded high sequence similarity (>98%) to UPWRP_2. This finding suggests that many of the *Haliangium* species present in the activated sludge ecosystem are not represented in the SILVA database. It reiterates the need for a sample-centric database for probe and primer design, and to constantly evaluate the environmental specificity of the FISH probe against a sample-specific genomic database.^11^

Amplification and representation bias catalysed from the use of MDA^37^ also affects completeness of the genomic bins and presents challenges for the recovery of whole genomes.^13,38^ In our analysis, contamination in genomic bins were screened using reference-free methodology that were developed for SCG context.^39^ An analysis of the essential single-copy genes in the “mini-metagenome” of UPWRP_1 revealed the presence of two genomes which could not be deconvoluted using current taxonomy-independent binning algorithms.

It is important that the designed R-Probe is as specific as possible for its intended target OTU to: (1) reduce genomic heterogeneity prior to genomic assembly and (2) increase the completeness of the genomic bins. Given the short length of the ribotag template (33 bp), designing a specific probe is challenging. The following three approaches could be used in future experiments to increase the specificity of R-Probes.

The first approach involves using the entire 33 bp ribotag as an R-Probe. In most instances, an OTU is defined by its unique 33 bp ribotag sequence and the OTU represents a substantial fraction of the longer representative sequence of the ribotag (Supplementary Fig. 2). As part of our validation procedures, the length of the ribotag was truncated so that R-Probes could be used at a standardised hybridisation temperature with published FISH probes. Truncation of the length of ribotags altered the probe specificity as many OTUs that contained the complementary binding site of the truncated FISH probe were included into the sorted samples. For example, the relative abundance of V6 sequence tags in the “mini-metagenome” of probe Ribo_Thau1029_17 that matched to the 17 bp ribotag (98.97% ± 4.00) and 33 bp ribotag (93.79% ± 12.20) was different. A total of 33/66 of V6 sequence tags in the sorted samples for Ribo_Thau1029_17 contained the FISH probe sequence, and was co-sorted together with the intended *Thauera* OTU. Only one sequence tag could be matched to the 33 bp ribotag.

The inclusion of closely related OTUs in the genomic assembly, coupled with a low-sequencing depth used resulted in the *Thauera* bin having a genome completeness of 39%. A low-sequencing depth was used for the *Thauera* case study as the primary goal was a proof of principle experiment. In contrast, the relative abundance of V6 sequence tags in the “mini-metagenome” of probe Ribo_Unk1029_17 that matched to 17 bp ribotag (99.87% ± 0.28) and 33 bp ribotag (99.67% ± 0.48) was similar. Therefore, Ribo_Unk1029_17 captured majority of its target OTU. Coupled with a higher depth of sequencing, a higher completeness (98%) of the genomic bin of UPWRP_1 was attained.

The second approach involves designing multiple FISH probes for the target OTU. A longer representative sequence (81 bp) could be retrieved for the respective ribotag using the RiboTagger software (Supplementary Fig. 1). Subsequently, comparative sequence analysis of the representative sequence of the target OTU and other non-target OTUs in the sequencing dataset allows FISH probes to be designed with central mismatch to other non-target OTUs, thus increasing the specificity of the FISH probe.

The final approach involves designing FISH probes from other variable regions of the 16S rRNA gene of the same target taxon. This can be achieved using the RiboTagger software to extract other variable regions (V4, V5 and V7). However, this approach requires the target OTU to have an established taxonomy for probes to be design against the same target organism. Alternatively, FISH probes from other hypervariable regions can be designed using the approach of Hasegawa et al.,^14^ where full-length 16S rRNA references sequences containing the V6 sequence is downloaded from a curated database for probe design with comparative sequence analysis. However, there is a risk that the target OTU in the sample might have a different full-length 16S rRNA sequence from the curated database.

Finally, the spatial resolution provided by R-Probes would yield additional ecological information that could complement the community profiling and functional analysis provided by metagenome and/or metatranscriptome analyses. Analysing the intra-community spatial architecture of target groups could reveal tight biological interactions between groups of cells such as the mutualistic symbiosis between ammonia- and nitrite-oxidising bacteria,^40^ or mutual avoidance, consistent with our data from UPWRP_2 and the other *Haliangium* sp. One of the discoveries through FISH visualisation is the suggestion of species-specific aggregation of UPWRP_2. In this study, the spatial structure of the *Haliangium* OTUs differed from other *Haliangium* sp. documented in wastewater treatment systems.^41^ *Haliangium* sp. can be classified under the Myxobacteria—a group of bacteria that forms fruiting bodies as part of its multicellular lifestyle as observed from axenic culture studies.^42^ This study outlines an integrated methodology for performing targeted characterisation of a specific OTU in a sample, especially in the scenario where no canonical probe or full-length 16S rRNA sequences are available for FISH probe design.

## Methods

A flowchart of the methods is presented in Supplementary Fig. 12.

### Bacterial culture and growth conditions

*Thauera*, a dominant member (2.88% in the metatranscriptomics dataset) of the activated sludge community in a wastewater treatment plant in Singapore treating mostly municipal effluent, was used as a reference taxonomic group. Currently, only one canonical FISH probe: Thau646^43^ is known to be highly specific for *Thauera*. An axenic culture of *Thauera*, *Thauera* sp. R086 (accession number: KC252920) that was isolated directly from the wastewater plant was used as the reference organism for the validation of R-Probe. *Thauera linaloolentis* DSM 12138 (DSMZ, Germany) was used as the non-target organism for the calibration of probe Ribo_Thau1029_17. The cultures were grown aerobically in Luria–Bertani media, in a shaking incubator with a rotation speed of 200 rpm at 30 °C for 12 h.

### Acquisition and processing of activated sludge samples

Activated sludge was sampled and transported to the laboratory on ice, followed by storage at 4 °C until sample processing. The vortexed sample was aliquoted as follows: 100 µL for FISH–FACS experiments; 1 mL for fixation and 2 mL for total genomic DNA extraction. The number of biological and technical replicates used for experiments are presented in Supplementary Table 9.

### Sample fixation for FISH visualisation

In situ visualisation was performed on paraformaldehyde-fixed samples. Both axenic cultures and sludge samples were fixed using the same protocol.^44^

### Genomic DNA extraction

DNA was extracted from pre-sorted samples using the FastDNA SPIN KIT for Soil (MP Biomedicals, USA), where homogenisation was performed for four cycles of 40 s at a speed of 6.0 m/s using a FastPrep-24 bead homogeniser (MP Biomedicals, USA). Eluted DNA was further purified using genomic DNA Clean & Concentrator (Zymo Research, USA).

### Optimising parameters for fluorescent in situ hybridization (FISH)

A standardised hybridisation temperature (46 °C) and washing temperature (48 °C) were used for all FISH analysis.^19^ Optimal hybridisation stringency of newly developed probes was determined using a formamide dissociation curve. Dissociation curve was generated from technical triplicates, where mean fluorescence intensities of probe-labelled objects in multiple field-of-views (*n* = 12) were acquired at a formamide concentration range of 10–70% using a microscope (Zeiss LSM 780, Germany). Images were imported into the Digital Image Analysis In Microbial Ecology software,^45^ where probe-labelled objects were segmented using the ‘RATS-L’ algorithm. Mean fluorescence intensities were plotted against each respective formamide concentration in a melting curve.

### Microscopic imaging

Fixed samples were immobilised on microscopic slides (eight wells with 6 mm diameter, Cell-Line, USA) by drying in the hybridisation oven (Shake ‘n’ Stack, Thermo Fisher, USA). Slides were subsequently dehydrated in an ethanol (Merck, USA) dehydration series: 50%, 80% and 96% (v/v) for 3 min, respectively. Hybridisation buffer was added to the sample, followed by the addition of FISH probes to a final concentration of 5 ng/µL. Optimal hybridisation and washing condition were based on the melting curve of the FISH probe (Supplementary Table 10).^46^ Samples were examined through a confocal laser scanning microscope (Zeiss LSM 780, Germany).

### Image analysis: quantitative FISH

Quantitative FISH analysis was performed to quantify the relative abundance of the target taxon through the following equation:

1 $$\begin{array}{l}{\mathrm{Relative}}\,{\mathrm{abundance}}\,{\mathrm{of}}\,{\mathrm{target}}\,{\mathrm{taxon}}= \frac{{{\mathrm{biovolume}}\,{\mathrm{of}}\,{\mathrm{target}}\,{\mathrm{taxon}}\,{\mathrm{hybridised}}\,{\mathrm{by}}\,{\mathrm{specific}}\,{\mathrm{probe}}}}{{{\mathrm{biovolume}}\,{\mathrm{of}}\,{\mathrm{biomass}}\,{\mathrm{hybridised}}\,{\mathrm{by}}\,{\mathrm{probe}}\,{\mathrm{EUB}}338}}\end{array}$$

Biovolume was calculated with the Imaris 8.2.0 software (Bitplane, Switzerland). To obtain the biovolume, multiple 3D microscopic images in random positions were acquired. Probe-labelled cells were segmented using the ‘surface segmentation’ algorithm. Subsequently, a filter was used to remove background noise on the segmented images by using an absolute intensity threshold value of >10.

### Image analysis: Manders’ co-localisation coefficient

Manders’ co-localisation coefficient (MCC) was obtained after acquisition of multiple fields-of-view of images (*n* = 22). Two channels, which represented the Cy5 and Cy3 fluorophores attached to probes Ribo_Thau1029_17 and Thau646 respectively, were selected for co-localisation analyses with Imaris 8.2.0 software (Bitplane). MCC values were derived from the ‘co-localisation’ function of the Imaris software. MCC estimates the contribution of Cy5 and Cy3 to the co-localised regions, and it has values ranging from 0 to 1: a value of 1 indicates that 100% of the channel will co-localise and a value of 0 indicates that none of the channels will co-localise.

### Fixation-free, in-solution FISH for FACS

Fixation-free in-solution FISH^29^ was performed prior to cell sorting so that sorted samples were amenable to downstream genomic sequencing and analysis. *Thauera* and UPWRP_2 aggregates were disintegrated through sonication performed with a sonicator (SONICS Vibracell VCX 750 Ultrasonic Cell Disrupter, USA) for 15 s with an interval of 5 s pulse. Aggregates of UPWRP_1 were broken up by parsing the hybridised sample repeatedly through a syringe needle (26G × ½″, Terumo, Japan). The efficiency of both procedures was verified through microscopy (Zeiss LSM 780, Germany).

### FACS

Prior to cell sorting, FACS clean solution (BD Biosciences, USA) was parsed through the fluidic lines of the sheath- and sampling-port of the FACS machine for 1 h, followed by 30 min of flushing with DNA-free water. New sterile sheath fluid (IsoFlow Sheath Fluid, Beckman Coulter, USA) was used for each sorting experiment. These procedures helped in sterilising the FACS machine and minimising the entry of contaminants that would complicate downstream MDA reactions.

Flow sorting and cytometric analysis were performed on the MoFlo XDP (Beckman Coulter, USA) that was operated with a 100 μm nozzle and 30 psi sheath liquid pressure. A 488 nm solid state laser (100 mW) was used as an excitation source for the measurement of Alexa 488 fluorophore and light-scattering properties, and a 640 nm laser (20 mW) was used as an excitation source for the measurement of Cy5 fluorophore. The MoFlo XDP sorter was equipped with a Nanoview small particle detection module (Propel Labs, Fort Collins, USA) and calibrated with 0.4 μm and 0.8 μm beads.

Forward scatter was detected with the Nanoview module; side scatter was detected with a BP 488/10; Alexa 488 fluorescence was detected with BP 530/30 and Cy5 fluorescence was detected with BP 670/40. The drop delay was calibrated using fluorescent FlowCheck pro beads (Beckman coulter) and ‘auto drop delay wizard’ in Summit software (Beckman Coulter). Analysis of data on the actual sorting days was performed with the Summit software. Post hoc flow cytometric plots were presented using the FlowJo software (LLC, USA).

Four sorting gates were constructed to exclude smaller cellular aggregations and to select for probe-labelled cells that displayed fluorescence intensity above the negative hybridisation controls. The controls were: (1) no-probe control where no FISH probes were added and (2) non-specific controls where probe NON338 was labelled with either a Cy5 fluorophore or Alexa 488 fluorophore to estimate the levels of non-specific binding for the respective fluorophores.

Sorting gate 1 differentiates bacterial cells on their forward and side light-scattering properties. Big cellular aggregates were filtered out through sorting gates 2 and 3, which was constructed on the assumption that a single cell shared a linear relationship between the forward/side scatter height and area. In contrast, forward/side scatter height of cellular aggregates will remain the same, but the forward/side scatter area will be larger than that of single cell. Sorting gate 4 was constructed to isolate probe-labelled cells exhibiting high Cy5 and A488 fluorescence over the other non-labelled cells or background noise.

Two rounds of sorting were performed with the defined sorting gates. In the initial round of sorting, ~150,000 events were sorted with ‘high purity’ mode into a sterile tube containing 1 mL of TE buffer. Subsequently, a second round of sorting with ‘single-cell’ mode was performed on the initial sorted cells and cells were sorted into sterile tubes containing 3 µL of TE buffer. Sorting efficiency was verified by visualising the sorted cells with a microscope.

### Multiple displacement amplification (MDA)

Genomic sequences of the sorted cells were amplified with whole genome amplification (WGA) to meet the required minimum concentration of 20 ng/µL for Illumina genome sequencing. Cell lysis of sorted cells and WGA was performed using MDA (REPLI-g single cell kit, Qiagen, USA). Amplified DNA was purified using ethanol precipitation as described by the QIAGEN REPLI-g single cell supplementary protocol: purification of DNA amplified using REPLI-g kits.

### Construction of full-length 16S rRNA gene clone library

Full-length 16S rDNA clone libraries were constructed from a single-sorted, MDA-purified sample for phylogenetic analyses. PCR amplification was performed using the universal bacterial primer pair 27F/1492R and *Taq* polymerase with the following settings: initial denaturation at 95 °C for 3 min, followed by 30 cycles of denaturation at 95 °C for 30 s, annealing at 55 °C for 30 s, extension at 72 °C for 2 min and a final extension step at 72 °C for 10 min. PCR amplicon products were verified with 1% agarose gel (w/v) and purified using the PureLink^®^ PCR purification kit (Life Technologies, USA).

A clone library was generated using the TOPO TA cloning kit (Invitrogen, USA). A total of 60 clones were selected and the plasmids were extracted using the PureLink Quick Plasmid Miniprep Kit (Invitrogen, USA). Plasmids were sequenced on the Applied Biosystems 3730xl DNA analyser (First Base, Singapore) using the primer sets of M13F (-20) and M13R-pUC-(-26). Trimming of low-quality sequences was performed before assembling the forward and reverse reads of individual clones with the SeqMan Pro software (DNASTAR, USA). Only clones with near full-length sequences (≥1200 bp) were selected. Presence of chimeric sequences was screened using the DECIPHER web-interface tool.^47^

### Full-length 16S rRNA gene analysis

Full-length 16S rDNA sequences of clones were clustered into OTUs using a 99% similarity cut-off value using the pick_otus.py script in QIIME.^48^ Representative sequences were selected from the most abundant sequence of the OTUs using the pick_rep_set.py script in QIIME, and subsequently taxonomically classified with SINA Alignment tool^49^ using the lowest common ancestor method. SINA was also used to: (1) perform sequence alignment; (2) retrieve closely related 16S rRNA gene sequences from the SILVA database and (3) calculate pairwise sequence similarity between 16S rRNA gene sequences. Representative sequences and its closely neighbours, together with the curated SILVA 123 SSU Ref NR99 database were imported into the ARB software.^50^ Alignment was manually inspected in ARB edit. A maximum-likelihood tree was constructed using the randomized axelerated maximum likelihood program with rapid bootstrap analysis of 1000 iterations.

### RiboTagger 16S rRNA analysis

Taxonomic profiling was performed using RiboTagger.^16^ Reads originating from the V6 regions of the 16S rRNA gene were extracted and taxonomically classified using the SILVA database. Relative abundance of a taxon was derived through the following equation:

2 $${\mathrm{Relative}}\,{\mathrm{abundance}}\,{\mathrm{of}}\,{\mathrm{a}}\,{\mathrm{taxon}} = \frac{{{\mathrm{Sum}}\,{\mathrm{of}}\,{\mathrm{sequence}}\,{\mathrm{tags}}\,{\mathrm{annotated}}\,{\mathrm{to}}\,{\mathrm{the}}\,{\mathrm{taxon}}}}{{{\mathrm{Total}}\,{\mathrm{number}}\,{\mathrm{of}}\,{\mathrm{sequence}}\,{\mathrm{tags}}\,{\mathrm{in}}\,{\mathrm{the}}\,{\mathrm{sample}}}}$$

### Metagenomic sequencing and library construction

Illumina TruSeq Nano DNA sample preparation protocol was used for sequencing library preparation. Genomic sequencing of *Thauera* samples was performed on a MiSeq (Illumina, USA); genomic sequencing of UPWRP_1 and UPWRP_2 samples was performed on a HiSeq 2500 (Illumina, USA). Adaptors were removed, and reads were quality trimmed with a minimum Phred score of 20 and a minimum length of 30 bp from both ends using the software BBDuk tools (BBMap–Bushnell B. http://sourceforge.net/projects/bbmap).

### Metagenomic assembly and quality-processing of contigs

Quality-trimmed reads were assembled using the SPAdes software^51^ with the following settings: sc and careful. Contigs shorter than 1 Kbp were removed. Remaining contigs were screened for contaminants using the Acdc software^39^ with the default settings.

### Estimation of genome completeness, contamination levels and sequencing coverage

Completeness and contamination levels of genomic bins were estimated through the CheckM software,^52^ which estimates the proportion of lineage-specific marker genes. Completeness and contamination level were further validated by estimating the fraction of essential single-copy genes conserved in 95% of bacteria^53^ using the Anvi’o software.^54^ Average sequencing coverage of samples was estimated using BBMap tool with the default setting (BBMap–Bushnell B. http://sourceforge.net/projects/bbmap).

### Extraction of 16S rRNA gene sequences from genomic bin

16S rRNA gene sequences present in genomic bin were retrieved using the rRNA.sh script from the mmgenome toolbox.^55^ Extracted 16S rRNA gene sequences were aligned and taxonomically classified using the SINA software.

### Metagenomic binning

A cluster of contigs extracted by the Acdc software was subjected to further metagenomic binning using the MetaBat software.^56^ TNF only binning was applied, and draft genomes recovered were examined for completeness and contamination with CheckM software.

### Phylogenomic analysis of draft genomes

Draft genomes were phylogenetically assigned using a concatenated set of phylogenetic conserved marker genes and placed in the reference genome tree from the IMG database using the CheckM software. Phylogenetic relationship of the draft genomes was presented using the ARB software.^50^

### Reporting summary

Further information on experimental design is available in the Nature Research Reporting Summary linked to this paper.

## Supplementary information

Supplementary information

Reporting summary

## Data Availability

The data supports the results reported in this study and its supplementary information. Near full-length 16S rDNA sequences obtained from the clone libraries of UPWRP_1 and UPWRP_2 were deposited in NCBI’s GenBank database with the following accession numbers: KX907544-KX907601 and KX954221-KX954279, respectively. Draft genomes of UPWRP_1, UPWRP_2 and *Thauera* have been deposited in NCBI under Bioproject accession IDs: PRJNA437784, PRJNA359471 and PRJNA503515, respectively. Other data that support the findings of this study are available from the corresponding author on reasonable request. Supplementary information is available at npj Biofilms and Microbiomes website.

## Supplementary information

**Supplementary information** accompanies the paper on the *npj Biofilms and Microbiomes* website (10.1038/s41522-019-0090-9).
