# Supplementary material for: Primer-free FISH probes from metagenomics/metatranscriptomics data permit the study of uncharacterised taxa in complex microbial communities
Source: NPJ Biofilms Microbiomes. 2019 Jun 25;5:17. doi: 10.1038/s41522-019-0090-9 (PMC6592924; doi:10.1038/s41522-019-0090-9)
Supplement: Supplementary file 1 — Supplementary information [file 41522_2019_90_MOESM1_ESM.pdf]

Supplementary Table 1: Homologous probe binding site of Ribo_Thau1029_17 in *Thauera* sp. R086 and *Thauera Linaloolentis*

Organisms	Probe target site
R086 (accession number: KC252920)	TTCGGGAGCC**A**G**A**ACAC
Thauera linaloolentis (DSM 12138)	TTCGGGAGCC**T**G**G**ACAC

Mismatched regions are highlighted in bold

Supplementary Table 2: Sequencing information of pre- and post-sorted samples

Sample type	R-Probe	Target taxon	No of sorted samples used for genomic assembly	No of paired-end reads (passed QC)	Sequencing platform	Read-length	Size of assembly (Mbp) *	% of reads matching OTU V6 sequence	% of reads matching R-Probe	Average coverage to *Thauera* genomic bin	Average coverage to UPWRP_1 genomic bin	Average coverage to UPWRP_2 genomic bin
Pre-sorted samples	NA	NA	9/9	5,543,160	Mi-seq	2 x 300bp	40.57	0	0	2.97 ± 7.54	NA	NA
Sorted-samples	Ribo_Thau1029_17	*Thauera*	27/32^	27,744,702	Mi-seq	2 x 300bp	7.94	93.79% ± 12.20	98.97% ± 4.00	270.20 ± 2227.65	NA	NA
Pre-sorted samples	NA	NA	6/6	74,527,740	Hi-seq	2 x 250bp	NA@	0	0	NA	0.57 ± 6.13	NA
Sorted-samples	Ribo_Unk1029_17	UPWRP_1	4/16#	48,339,304	Hi-seq	2 x 250bp	12.70	99.67% ± 0.48	99.87% ± 0.28	NA	525.85 ± 594.72	NA
Pre-sorted samples	NA	NA	6/6	64,047,240	Hi-seq	2 x 250bp	NA@	0	0	NA	NA	19.96 ± 50.44
Sorted-samples	Ribo_Halia1029_17	UPWRP_2	1	14,008,892	Hi-seq	2 x 250bp	2.42	100	100	NA	NA	1182.24 ± 8889.18

NA Not available

NA@ Not available due to computational limitation of using the SPAdes assembler software

Only 4 samples were selected for co-assembly due to computational limitation of using the SPAdes assembler software

^ Only samples with more than 90% of the V6 sequence tag matching the targeted OTU were selected for genomic assembly

* Assembly only contain contigs ≥ 1000 bp in length

Supplementary Table 3: RiboTagger 16S rRNA analysis of samples sorted with Ribo_Thau1029_17

Tag sequence	No of sequence tags	Representative sequence	Annotation
GTGTTCTGGCTCCCGAAGGCACCCTCGCCTCTC	31651	GACGACAGCCATGCAGCACCTGTGTTCTGGCTCCCGAAGGC<br>ACCCTCGCCTCTCAGCAAGGTTCCAGACATGTCAAGGGTA	k_Bacteria; p_Proteobacteria; c_Betaproteobacteria; o_Rhodocyclales; f_Rhodocyclaceae; g_Thauera
GTGTTCTGGCTCCCGAAGGCACCCTCGGCTCTC	246	GACGACAGCCATGCAGCACCTGTGTTCTGGCTCCCGAAGGC<br>ACCCTCGGCTCTCACCAAGGTTCCAGACATGTCAAGGGTA	
GTGCTCTGGCTCCCGAAGGCACCCTCGCCTCTC	2	GACGACAGCCATGCAGCACCTGTGCTCTGGCTCCCGAAGGC<br>ACCCTCGCCTCTCAGCAAGGTTCCAGACATGTCAAGGGTA	
GTGTTCTGGCTCCCGAAGGCACTCCCCGATCTC	215	GACGACAGCCATGCAGCACCTGTGTTCTGGCTCCCGAAGGC<br>ACTCCCCGATCTCTCAGGGATTCCAGACATGTCAAGGGTA	
GTGTTCCGGTTCTCTTACGAGCACTCCTAAATC	79	GACGACAGCCATGCAGCACCTGTGTTCCGGTTCTCTTACGA<br>GCACTCCTAAATCTCTTCAGGATTCCAGACATGTCAAGGG	
GTGTTCTGGCTCCCGAAGGCAGCCCCCTGGGCC	69	GACGACAGCCATGCAGCACCTGTGTTCTGGCTCCCGAAGGC<br>AGCCCCCTGGGCCTGTACTGACGCTCATGCACGAAAGCGT	
GTGTTCTGGCTCCCGAAGGCATAGACAGCTAGG	51	GACGACAGCCATGCAGCACCTGTGTTCTGGCTCCCGAAGGC<br>ATAGACAGCTAGGAGGTTGGCTTAGAAGCAGCCATCCTTT	
GTGTTCTGACTCCCGAAGGCACCCTCGCCTCTC	23	GACGACAGCCATGCAGCACCTGTGTTCTGACTCCCGAAGGC<br>ACCCTCGCCTCTCAGCAAGGTTCCAGACATGTCAAGGGTA	
GTGTTCTGGCTCCCGAAGGCACCCTCGCGTAGA	3	GACGACAGCCATGCAGCACCTGTGTTCTGGCTCCCGAAGGC<br>ACCCTCGCGTAGAGGTTTGCTCTTTAACAAAGTGGAAGAA	
GTGTTCTGGCTCCCGAAGACACCCTCGCCTCTC	18	GACGACAGCCATGCAGCACCTGTGTTCTGGCTCCCGAAGAC<br>ACCCTCGCCTCTCAGCAAGGTTCCAGACATGTCAAGGGTA	
GTGTTCTGGTTCCCGAAGGCACCCTCGCCTCTC	12	GACGACAGCCATGCAGCACCTGTGTTCTGGTTCCCGAAGGC<br>ACCCTCGCCTCTCAGCAAGGTTCCAGACATGTCAAGGGTA	
GTGTTCTGGCTCCCTTGCGGGCACCCCCGGCTT	17	GACGACAGCCATGCAGCACCTGTGTTCTGGCTCCCTTGCGG<br>GCACCCCCGGCTTTCACCAGAGTTCCAGACATGTCAAGCC	k_Bacteria; p_Cyanobacteria; c_Cyanobacteria; o_SubsectionIII; f_FamilyI; g_Leptolyngbya
GTGTTCTGGCGAGAAGAGCCTCGGATGAGGGTC	26	GACGACAGCCATGCAGCACCTGTGTTCTGGCGAGAAGAGCC<br>TCGGATGAGGGTCTGTAGCTCAGCTGGTTAGAGCACCGTC	
GTGTTCTAGCTCCCGAAGGCACCCTCGCCTCTC	10	GACGACAGCCATGCAGCACCTGTGTTCTAGCTCCCGAAGGC<br>ACCCTCGCCTCTCAGCAAGGTTCCAGACATGTCAAGGGTA	
GTGTTCTGGCACCCGAAGGCACCCTCGCCTCTC	10		
GTGTTCTGGCTCCCGAAGGCACCCTCGTCTCTC	8	GACGACAGCCATGCAGCACCTGTGTTCTGGCTCCCGAAGGC<br>ACCCTCGTCTCTCAGCAAGGTTCCAGACATGTCAAGGGTA	
GTGTTCTGGCTCCCGAAGGCACCCTTGCCTCTC	6		
GTGTTCTGGCTCCCGAAGGCACCCTCGCCGTAA	6	GACGACAGCCATGCAGCACCTGTGTTCTGGCTCCCGAAGGC<br>ACCCTCGCCGTAAGCCCAAGGTTTCCTGCGCAACGTTCAT	
GTGTTCTGGCTCCCGAGGGCACCCTCGCCTCTC	7	GACGACAGCCATGCAGCACCTGTGTTCTGGCTCCCGAGGGC<br>ACCCTCGCCTCTCAGCAAGGTTCCAGACATGTCAAGGGTA	
GTGTTCTGGCTCCCGAAGGCCCCCTGGGCCTGT	7	GACGACAGCCATGCAGCACCTGTGTTCTGGCTCCCGAAGGC<br>CCCCTGGGCCTGTACTGACGCTCATGCACGAAAGCGTGGG	

GTGTTCTGGCTCCTGAAGGCACCCTCGCCTCTC	10	GACGACAGCCATGCAGCACCTGTGTTCTGGCTCCTGAAGGC ACCCTCGCCTCTCAGCAAGGTTCCAGACATGTCAAGGGTA	
GTGTTCTGGCTCCCGAAGGCATCCTCGCCTCTC	14	GACGACAGCCATGCAGCACCTGTGTTCTGGCTCCCGAAGGC ATCCTCGCCTCTCAGCAAGGTTCCAGACATGTCAAGGGTA	
GTGTTCTGGCTCCCGAAGGCACCCTCACCTCTC	12		
GTGTTCTGCCTCCCGAAGGCACCCTCGCCTCTC	9	GACGACAGCCATGCAGCACCTGTGTTCTGCCTCCCGAAGGC ACCCTCGCCTCTCAGCAAGGTTCCAGACATGTCAAGGGTA	
GTGTTCTGGCTTCCGAAGGCACCCTCGCCTCTC	8	GACGACAGCCATGCAGCACCTGTGTTCTGGCTTCCGAAGGC ACCCTCGCCTCTCAGCAAGGTTCCAGACATGTCAAGGGTA	
GTGTTCTGGCTCCCGAAAGCGTGGGGAGCAAAC	4	GACGACAGCCATGCAGCACCTGTGTTCTGGCTCCCGAAAGC GTGGGGAGCAAACAGGATTAGATACCCTGGTAGTCCACGC	
GTGTTCTGGCTCCCGAAGGCACCCTCGCCTCTA	4		
GTGTTCTGGCTCCCGAAGGCACCCCCGCCTCTC	5	GACGACAGCCATGCAGCACCTGTGTTCTGGCTCCCGAAGGC ACCCCCGCCTCTCAGCAAGGTTCCAGACATGTCAAGGGTA	
GTACTGACGCTCATGCACGAAAGCGTGGGGAG C	5	GACGACAGCCATGCAGCACCTGTACTGACGCTCATGCACGA AAGCGTGGGGAGCAAACAGGATTAGATACCCTGGTAGTCC	
GTGTCCTGGCTCCCGAAGGCACCCTCGCCTCTC	2	GACGACAGCCATGCAGCACCTGTGTCCTGGCTCCCGAAGGC ACCCTCGCCTCTCAGCAAGGTTCCAGACATGTCAAGGGTA	
GTGTTCTGGCTCCCGAAGGCATGTGGTGGATGC	2	GACGACAGCCATGCAGCACCTGTGTTCTGGCTCCCGAAGGC ATGTGGTGGATGCCTTGGCGATCACAGGCGATGAAGGACG	
GTGTTCTGGCTCCCGAAGGCACCCTCGCCCCCT	2	GACGACAGCCATGCAGCACCTGTGTTCTGGCTCCCGAAGGC ACCCTCGCCCCCTGGGCCTGTACTGACGCTCATGCACGAA	
GTGTTCTGGCTCCCGAAGGCACCCTCGCCTGGG	4	GACGACAGCCATGCAGCACCTGTGTTCTGGCTCCCGAAGGC ACCCTCGCCTGGGGAGTACGGCCGCAAGGTTAAAACTCAA	
GTGTTCTGTCTCCCGAAGGCACCCTCGCCTCTC	2	GACGACAGCCATGCAGCACCTGTGTTCTGTCTCCCGAAGGC ACCCTCGCCTCTCAGCAAGGTTCCAGACATGTCAAGGGTA	
GTGTCCTGGCTCAGATTGAACGCTGGCGGCATG	3	GACGACAGCCATGCAGCACCTGTGTCCTGGCTCAGATTGAA CGCTGGCGGCATGCTTTACACATGCAAGTCGAACGGCAGC	
GCGTTCTGGCTCCCGAAGGCACCCTCGCCTCTC	6	GACGACAGCCATGCAGCACCTGCGTTCTGGCTCCCGAAGGC ACCCTCGCCTCTCAGCAAGGTTCCAGACATGTCAAGGGTA	
GTGTTCTGGCTCCCGAAGGCACCCTCGCCCCTC	5	GACGACAGCCATGCAGCACCTGTGTTCTGGCTCCCGAAGGC ACCCTCGCCCCTCAGCAAGGTTCCAGACATGTCAAGGGTA	
GTGTTCTGGCTCCCGAAGGCACCCTCGCCTCCC	5	GACGACAGCCATGCAGCACCTGTGTTCTGGCTCCCGAAGGC ACCCTCGCCTCCCAGCAAGGTTCCAGACATGTCAAGGGTA	
GTGTTCTGGCTCCCGAAAACTATTTAGGTAGTG	2	GACGACAGCCATGCAGCACCTGTGTTCTGGCTCCCGAAAAC TATTTAGGTAGTGCGTCGTACGGACACTTGCGGGGGTAGA	
GTGTTCTGGCTCCCGAAGGCACAGACAGCTAGG	2		
GTGTTCTGGCTCTCGAAGGCACCCTCGCCTCTC	6		
GTGTTCCGGCTCCCGAAGGCACCCTCGCCTCTC	7	GACGACAGCCATGCAGCACCTGTGTTCCGGCTCCCGAAGGC ACCCTCGCCTCTCAGCAAGGTTCCAGACATGTCAAGGGTA	k_Bacteria; p_Proteobacteria; c_Betaproteobacteria; o_Rhodocyclales; f_Rhodocyclaceae
GTGTTCTGGCTCCCGAAGGCGAAAAGAACCCCG	2	GACGACAGCCATGCAGCACCTGTGTTCTGGCTCCCGAAGGC GAAAAGAACCCCGGGAGGGGAGTGAAATAGATCCTGAAAC	

GTGTTCTGGCTCCCGAAGGCACCCACGCCTCTC	3	GACGACAGCCATGCAGCACCTGTGTTCTGGCTCCCGAAGGC ACCCACGCCTCTCAGCAAGGTTCCAGACATGTCAAGGGTA	
GTGTTCTGGCTCCCGAAGGCACCCTCGCCTATC	2	GACGACAGCCATGCAGCACCTGTGTTCTGGCTCCCGAAGGC ACCCTCGCCTATCAGCAAGGTTCCAGACATGTCAAGGGTA	
GTGTTCTGGCTCCCGAAGGTACCCTCGCCTCTC	2	GACGACAGCCATGCAGCACCTGTGTTCTGGCTCCCGAAGGT ACCCTCGCCTCTCAGCAAGGTTCCAGACATGTCAAGGGTA	
GAGTTCTGGCTCCCGAAGGCACCCTCGCCTCTC	3		
GTGTTCTGGCTCCAGAAGGCACCCTCGCCTCTC	4	GACGACAGCCATGCAGCACCTGTGTTCTGGCTCCAGAAGGC ACCCTCGCCTCTCAGCAAGGTTCCAGACATGTCAAGGGTA	
GTGTTCTGGCTACCGAAGGCACCCTCGCCTCTC	2	GACGACAGCCATGCAGCACCTGTGTTCTGGCTACCGAAGGC ACCCTCGCCTCTCAGCAAGGTTCCAGACATGTCAAGGGTA	
GGGTTCTGGCTCCCGAAGGCACCCTCGCCTCTC	2	GACGACAGCCATGCAGCACCTGGGTTCTGGCTCCCGAAGGC ACCCTCGCCTCTCAGCAAGGTTCCAGACATGTCAAGGGTA	
GTGTTCTGGCTCCGAAGGCACCCTCGCCTCTCA	2	GACGACAGCCATGCAGCACCTGTGTTCTGGCTCCGAAGGCA CCCTCGCCTCTCAGCAAGGTTCCAGACATGTCAAGGGTAG	
GTGTTCTGGCTCCCGAAGGTGGGGATGACGTCA	2	GACGACAGCCATGCAGCACCTGTGTTCTGGCTCCCGAAGGT GGGGATGACGTCAAGTCCTCATGGCCCTTATGGGTAGGGC	
GTGTTCTGGCTCCCGAAGGCACGTGGTAGGGGA	2	GACGACAGCCATGCAGCACCTGTGTTCTGGCTCCCGAAGGC ACGTGGTAGGGGAGCGTTCCGTAAGCCTGCGAAGGTGTCT	
GTGTGTGGTTTGCGCGGTACTCCTTACCCCTGA	2	GACGACAGCCATGCAGCACCTGTGTGTGGTTTGCGCGGTAC TCCTTACCCCTGACTCTTCACGAGTCGTGCTCTTTAACAA	
GTGTTCTGGCTCCCGAAGGCACCCTCGCCACTC	2	GACGACAGCCATGCAGCACCTGTGTTCTGGCTCCCGAAGGC ACCCTCGCCACTCAGCAAGGTTCCAGACATGTCAAGGGTA	
GTGTTCTGGCCCCCGAAGGCACCCTCGCCTCTC	3	GACGACAGCCATGCAGCACCTGTGTTCTGGCCCCCGAAGGC ACCCTCGCCTCTCAGCAAGGTTCCAGACATGTCAAGGGTA	
GTGTTCTGGCTCCCGAAGGCACCTTCGCCTCTC	2	GACGACAGCCATGCAGCACCTGTGTTCTGGCTCCCGAAGGC ACCTTCGCCTCTCAGCAAGGTTCCAGACATGTCAAGGGTA	
GTGTTGTTTCCCTCTTGACACCGGACGTTAGCA	2	GACGACAGCCATGCAGCACCTGTGTTGTTTCCCTCTTGACAC CGGACGTTAGCACCCGATGTCTGTCTGCCGTATATCACT	
GTGTTGGTTTGCGGTACGGTCACTCTTAGACTG	2	GACGACAGCCATGCAGCACCTGTGTTGGTTTGCGGTACGGT CACTCTTAGACTGAAGCTTAGAGGCTTTTCCTGGAAGCTG	
GTGTTCTGGCTCCCGAAGGAACCCTCGCCTCTC	2	GACGACAGCCATGCAGCACCTGTGTTCTGGCTCCCGAAGGA ACCCTCGCCTCTCAGCAAGGTTCCAGACATGTCAAGGGTA	
GTGTTCTGGCTCCCGAAGGCACCCTCGCCTTTC	2	GACGACAGCCATGCAGCACCTGTGTTCTGGCTCCCGAAGGC ACCCTCGCCTTTCAGCAAGGTTCCAGACATGTCAAGGGTA	
GTATTCTGGCTCCCGAAGGCACCCTCGCCTCTC	2	GACGACAGCCATGCAGCACCTGTATTCTGGCTCCCGAAGGC ACCCTCGCCTCTCAGCAAGGTTCCAGACATGTCAAGGGTA	
GTGTTCTGGCTCCCGATGGCACCCTCGCCTCTC	2	GACGACAGCCATGCAGCACCTGTGTTCTGGCTCCCGATGGC ACCCTCGCCTCTCAGCAAGGTTCCAGACATGTCAAGGGTA	
GTGTTCTGGCTCCCGAAGGCACCCTCGCCTCAC	2	GACGACAGCCATGCAGCACCTGTGTTCTGGCTCCCGAAGGC ACCCTCGCCTCACAGCAAGGTTCCAGACATGTCAAGGGTA	
GTGTTCTGGCTCCCAAAGGCACCCTCGCCTCTC	2	GACGACAGCCATGCAGCACCTGTGTTCTGGCTCCCAAAGGC ACCCTCGCCTCTCAGCAAGGTTCCAGACATGTCAAGGGTA	
GTGATCTGGCTCCCGAAGGCACCCTCGCCTCTC	2	GACGACAGCCATGCAGCACCTGTGATCTGGCTCCCGAAGGC ACCCTCGCCTCTCAGCAAGGTTCCAGACATGTCAAGGGTA	

NA Not available

Supplementary Table 4: Genome statistics of *Thauera*, UPWRP_1 and UPWRP_2

	Thauera	UPWRP_1	UPWRP_2
No of samples used for co-assembly	27	4	1
N50 (bp)	5,692	32,430	12,467
Minimum contig length (bp)	1,000		
Maximum contig length (bp)	71,687	145,517	55,295
GC content (%)	60.9	45.2	63.22
No of contigs	2,223	1,302	474

Supplementary Table 5: *In silico* coverage of Ribo_Halia1029_17 as predicted with the 'TestProbe' tool of the SILVA database

Probe sequence	Accession number of target organisms	Taxonomic classification
TCTCACTCGCTCCCGAA	AB286332	*Haliangium*
	AB286567	
	EU734997	

Supplementary Table 6: Major ribotags present in samples sorted with Ribo_Halia1029_17

Ribotags	Average relative abundance in sorted samples (%)	Annotation
**TCTCACTCGCTCCCGA**AGGCACCCCGACATCTC	44.14	UPWRP_2
**TCTCACTCGCTCCCGA**AGGCACCCCACCGTTTC	44.86	*Haliangium* species (accession no: AB286567)
GTGAACCGACCCCAAAAGAGGCACACCCATCTC	0.069	Propionibacterium
Other ribotags	10.93	No annotation

Sequence of probe Ribo_Halia1029_17 has been highlighted in bold. Sequence dissimilarities between the two ribotags of *Haliangium* have been underlined.

Supplementary Table 7: RiboTagger 16S rRNA analysis of samples sorted with Ribo_Unk1029_17

Tag sequence	No of sequence tags	Representative sequence	Annotation
TGCTTCGCGTCTCCGAAGAGCCGACCACCTTTC	6,289	GACGACAACCATGCAGCACCTTGCTTCGCGTCTCCGAAGAGCCGA CCACCTTTCAGCAGCCTCCGCTCGCATTCTAGCCCA	NA
TGCTTCGCGTCTCCGAAGAGCCGACCACCTTCT	2	GACGACAACCATGCAGCACCTTGCTTCGCGTCTCCGAAGAGCCGA CCACCTTCTTTCTACTTCTCTCTTTCCTGCCAGCAA	NA
TGCTTCGCGTCTCCGAAGAGTCGACCACCTTTC	2	GACGACAACCATGCAGCACCTTGCTTCGCGTCTCCGAAGAGTCGA CCACCTTTCAGCAGCCTCCGCTCGCATTCTAGCCCA	NA
TGCTTCGCGTCTCCGAAGATCCGACCACCTTTC	2	GACGACAACCATGCAGCACCTTGCTTCGCGTCTCCGAAGATCCGA CCACCTTTCAGCAGCCTCCGCTCGCATTCTAGCCCA	NA
TGCTTCGCGTCTCCGAAGAGCCGACCACCTTAC	2		NA
TGCTTCGCGTCTCCGAAGAGCTGACCACCTTTC	2	GACGACAACCATGCAGCACCTTGCTTCGCGTCTCCGAAGAGCTGA CCACCTTTCAGCAGCCTCCGCTCGCATTCTAGCCCA	NA
TGCTTCGCGTCTCCGAAGAGCCGACCACCATTC	2	GACGACAACCATGCAGCACCTTGCTTCGCGTCTCCGAAGAGCCGA CCACCATTCAGCAGCCTCCGCTCGCATTCTAGCCCA	NA
TGCTTCGCGTCTCCGAAGAGCCGACTACCTTTC	2	GACGACAACCATGCAGCACCTTGCTTCGCGTCTCCGAAGAGCCGA CTACCTTTCAGCAGCCTCCGCTCGCATTCTAGCCCA	NA
TGCTTCGTGTCTCCGAAGAGCCGACCACCTTTC	2	GACGACAACCATGCAGCACCTTGCTTCGTGTCTCCGAAGAGCCGA CCACCTTTCAGCAGCCTCCGCTCGCATTCTAGCCCA	NA
TCTCACTCGCTCCCGAAGGCACCCCGACATCTC	2	GACGACAGCCATGCAGCACCTTCTCACTCGCTCCCGAAGGCACCC CGACATCTCTGCCAGGTCCGAGTGGATTTAACCCAG	NA
TGCTTCGCGTCTCCGAGGAGCCGACCACCTTTC	2	GACGACAACCATGCAGCACCTTGCTTCGCGTCTCCGAGGAGCCGA CCACCTTTCAGCAGCCTCCGCTCGCATTCTAGCCCA	NA
TGCTTCATTTACAAACAAGGCAGACCGACTACC	2	GACGACAACCATGCAGCACCTTGCTTCATTTACAAACAAGGCAGA CCGACTACCTGTAGTAGTCCCGGGCAGACTTGAACT	NA
TGCTTCGCGTCCCCGAAGAGCCGACCACCTTTC	2	GACGACAACCATGCAGCACCTTGCTTCGCGTCCCCGAAGAGCCGA CCACCTTTCAGCAGCCTCCGCTCGCATTCTAGCCCA	NA
TGCTTCACGTCTCCGAAGAGCCGACCACCTTTC	2	GACGACAACCATGCAGCACCTTGCTTCACGTCTCCGAAGAGCCGA CCACCTTTCAGCAGCCTCCGCTCGCATTCTAGCCCA	NA
TGCTTCGCGTTACTCAGGTATCCAACCTCTAAA	2	GACGACAACCATGCAGCACCTTGCTTCGCGTTACTCAGGTATCCA ACCTCTAAAAGATTATTTACATGTACGGGGCTGTCA	NA

NA Not available

Supplementary Table 8: Sequence similarity between the 16S rRNA sequences of OTUs retrieved from UPWRP_1-enriched samples

	OTU1_Clone36	OTU2_Clone31	OTU3_Clone9	OTU4_Clone14
OTU1_Clone36	100%	98.92%	98.11%	98.97%
OTU2_Clone31		100%	98.92%	98.18%
OTU3_Clone9			100%	98.72%
OTU4_Clone14				100%

Supplementary Table 9: Biological and technical replicates of activated sludge samples used in various experiments

		Number of technical replicates		
Target taxon	Biological replicates and sampling date	DNA extraction*	FISH analysis*	FACS
Thauera	January 21st, 2016	3	3	1
	March 9th, 2016	3	3	1
	March 23rd, 2016	3	3	1
UPWRP_1	August 5th, 2016	3	3	1
	August 11th, 2016	3	3	1
UPWRP_2	August 23rd, 2016	3	3	1
	August 29th, 2016	3	3	1

*Performed on pre-sorted samples

Supplementary Table 10: Components of hybridisation and washing buffers used in FISH experiments

Supplementary Table 10.1: Components of hybridisation buffer

Components of hybridisation buffer	Volume (µL)	Final concentration in hybridisation buffer
5M NaCl	180	900 mM
1M Tris/HCL	20	20 mM
Formamide	Volume and concentration of formamide is dependent on probe stringency	
10% SDS	1	0.01%
MilliQ-water	Top up to a final volume of 1 ml	

Final volume of hybridisation buffer is 1mL. Volume of formamide to be added is dependent on the formamide concentration determined from melting curve analysis of FISH probes.

Supplementary Table 10.2: Components of washing buffer

Components of washing buffer	Volume (mL)	Final concentration in washing buffer
5M NaCl	Volume of NaCl is dependent on [formamide] used in hybridisation experiments	
1M Tris/HCL	1	20 mM
0.5M EDTA*	0.5	5 mM
MilliQ-water	Top up to a final volume of 50 mL	

* EDTA is only added if [formamide] is ≥20%.

Final volume of washing buffer is 50 mL. Volume of formamide to add is dependent on the formamide concentration determined from melting curve analysis of FISH probes.

Supplementary Table 10.3: Correlation between the concentration of formamide in hybridisation buffer and the concentration of NaCl in washing buffer

[Formamide] in hybridisation buffer	[NaCl] in washing buffer (M)	Volume of NaCl in 50 mL of washing buffer (µL)
0	0.900	9000
5	0.636	6300
10	0.450	4500
15	0.318	3180
20	0.225	2150
25	0.159	1490
30	0.112	1020
35	0.080	700
40	0.056	460
45	0.040	300
50	0.028	180
55	0.020	100
60	0.014	40
65	-	-
70	-	-

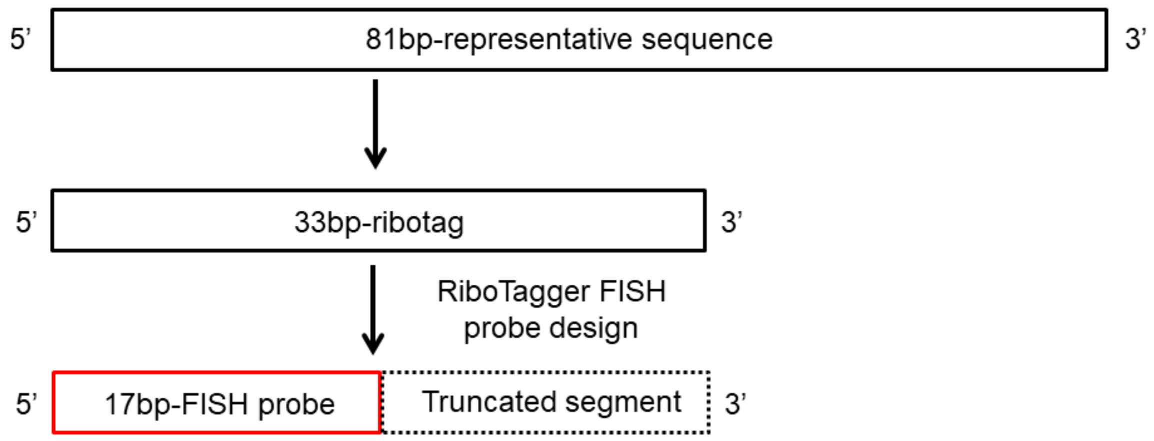

Supplementary Figure 1: The design of R-Probe from ribotag. Truncation of the length of the ribotag from the 3' end resulted in a 17bp-FISH probe.

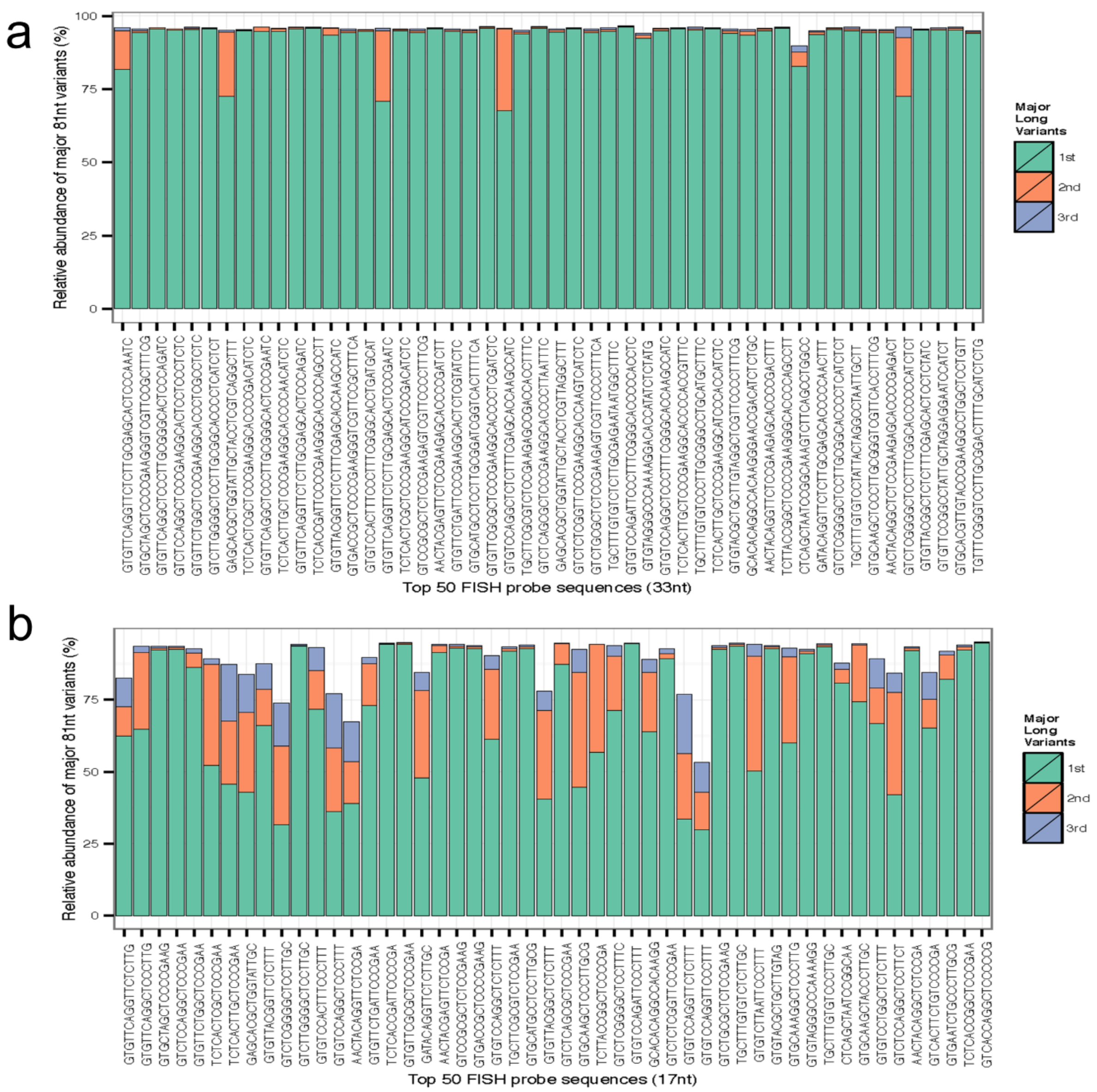

Supplementary Figure 2: *In silico* evaluation of the specificity of R-Probes at (a) 33 bp versus (b) 17 bp. Variants are defined as OTUs sharing identical FISH probe sequence, but having different representative sequences. A reduction in the length of R-Probe from 33 bp to 17 bp reduced the probe specificity for its target OTU, with more variants being hybridised by the probe. Data was obtained from the initial 16S rRNA sequence profiling.

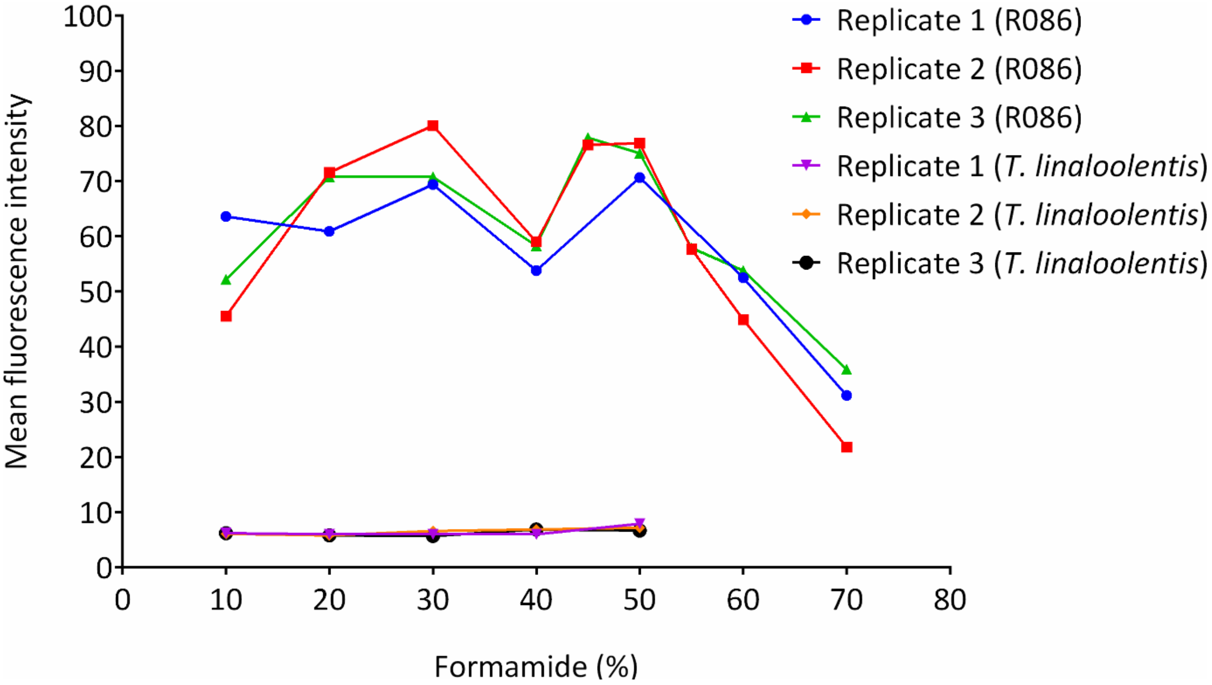

Supplementary Figure 3: Probe dissociation curve of Ribo_Thau1029_17. Probe dissociation curve was generated through a series of FISH experiments that were performed on an axenic culture of *Thauera* sp. R086 as the target organism. *Thauera linaloolentis* was used as the non-target organism.

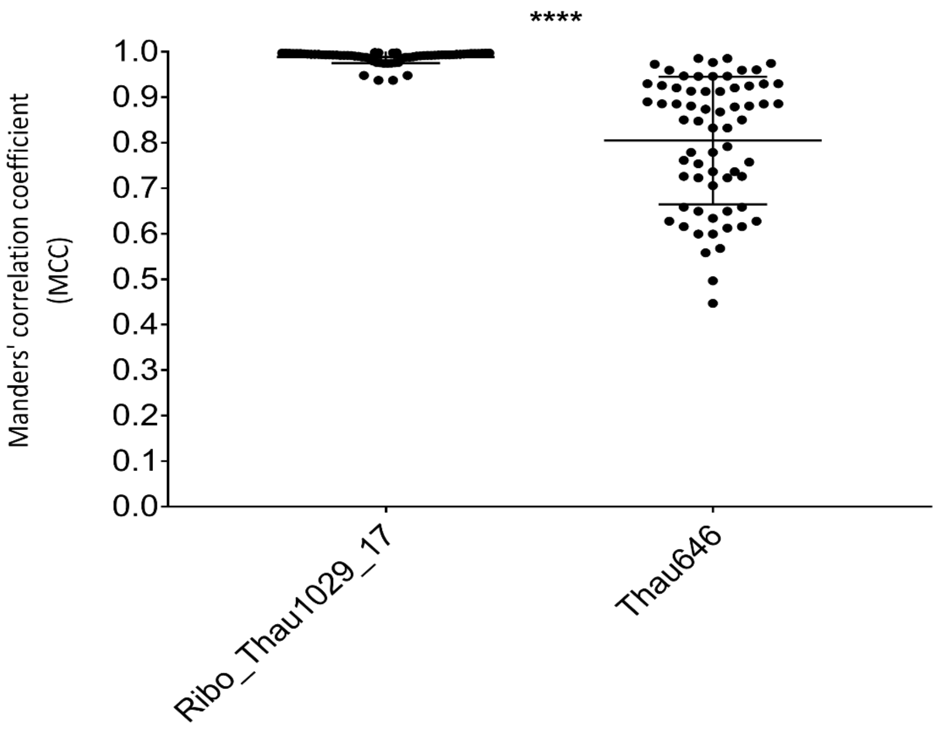

Supplementary Figure 4: Manders' co-localisation coefficient (MCC) obtained from quantitative co-localisation analysis. Activated sludge samples were co-hybridised with Ribo_Thau1029_17$_{Cy5}$ and Thau646$_{Cy3}$. Each dot represents MCC analysis performed on one image.

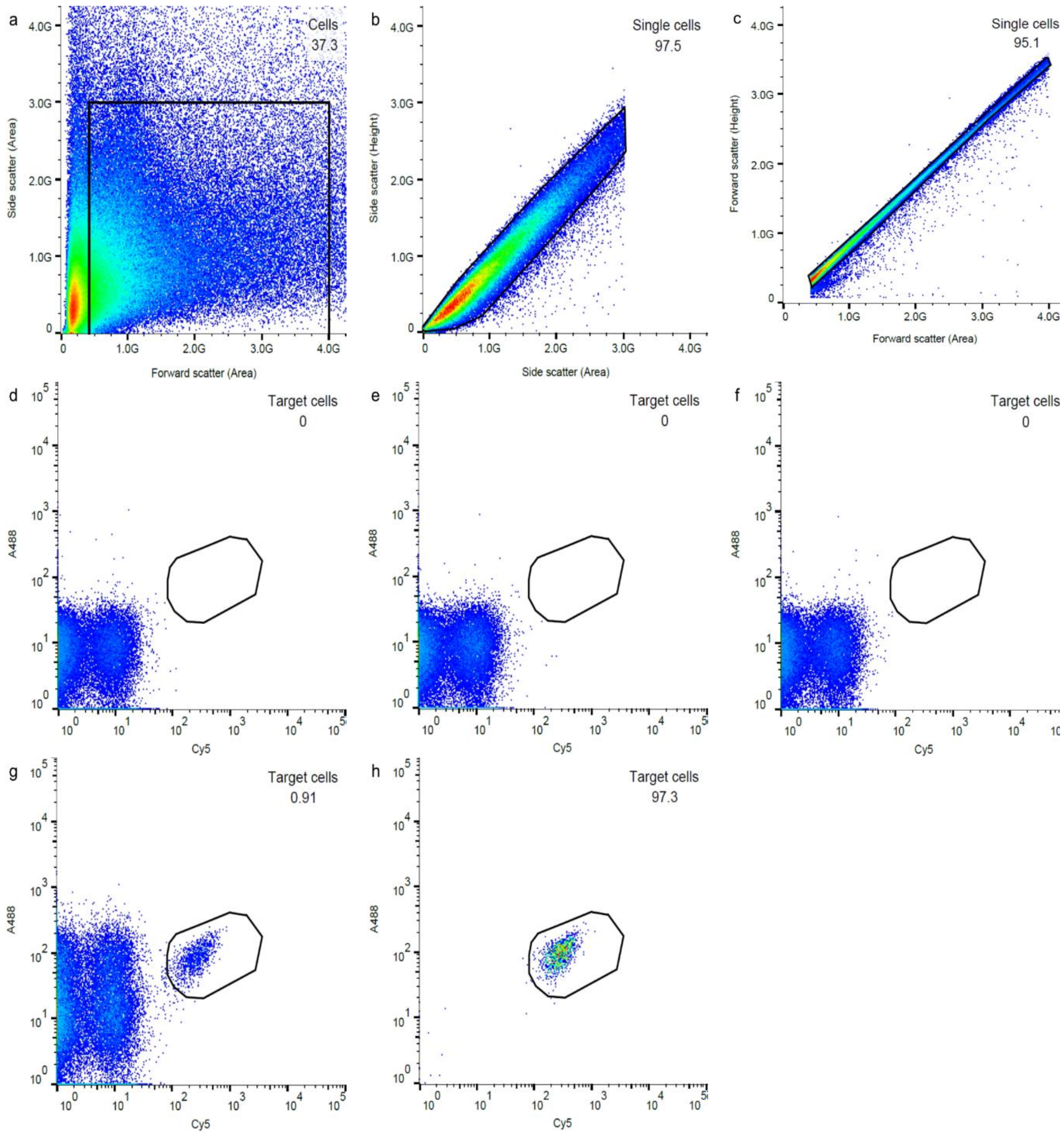

Supplementary Figure 5: Flow cytometric analysis of sorting *Thauera* from an activated sludge sample hybridised with probes Ribo_Thau1029_17$_{Cy5}$ and EUB388$_{A488}$.

An approximate ~100,000 events were collected for each FACS plot, except for the purity check. Sorting gates are outlined in black and values shown indicate the percentage of gated events over the total number of events. Flow cytometric analysis: (a) Sorting gate 1: sorting of bacterial cells using their light-scattering properties with a plot of forward versus side scatter; (b) sorting gate 2: filtering out cell aggregates based on side scatter area versus side scatter height; (c) sorting gate 3: filtering out cell aggregates based on forward scatter area versus forward scatter height. Sorting gate 4 was constructed to exclude events exhibiting Cy5 and A488 fluorescence signal in the negative controls: (d) no-probe control; (e) hybridisation with probe NON338$_{Cy5}$ to estimate non-specific binding for Cy5 fluorophore; (g) hybridisation with probe NON338$_{A488}$ to estimate non-specific binding for A488 fluorophore. (g) Events exhibiting Cy5 and A488 fluorescence signal above the cut-off threshold for the negative

controls were collected in sorting gate 4. (h) Purity of the sorted sample after an initial round of sorting.

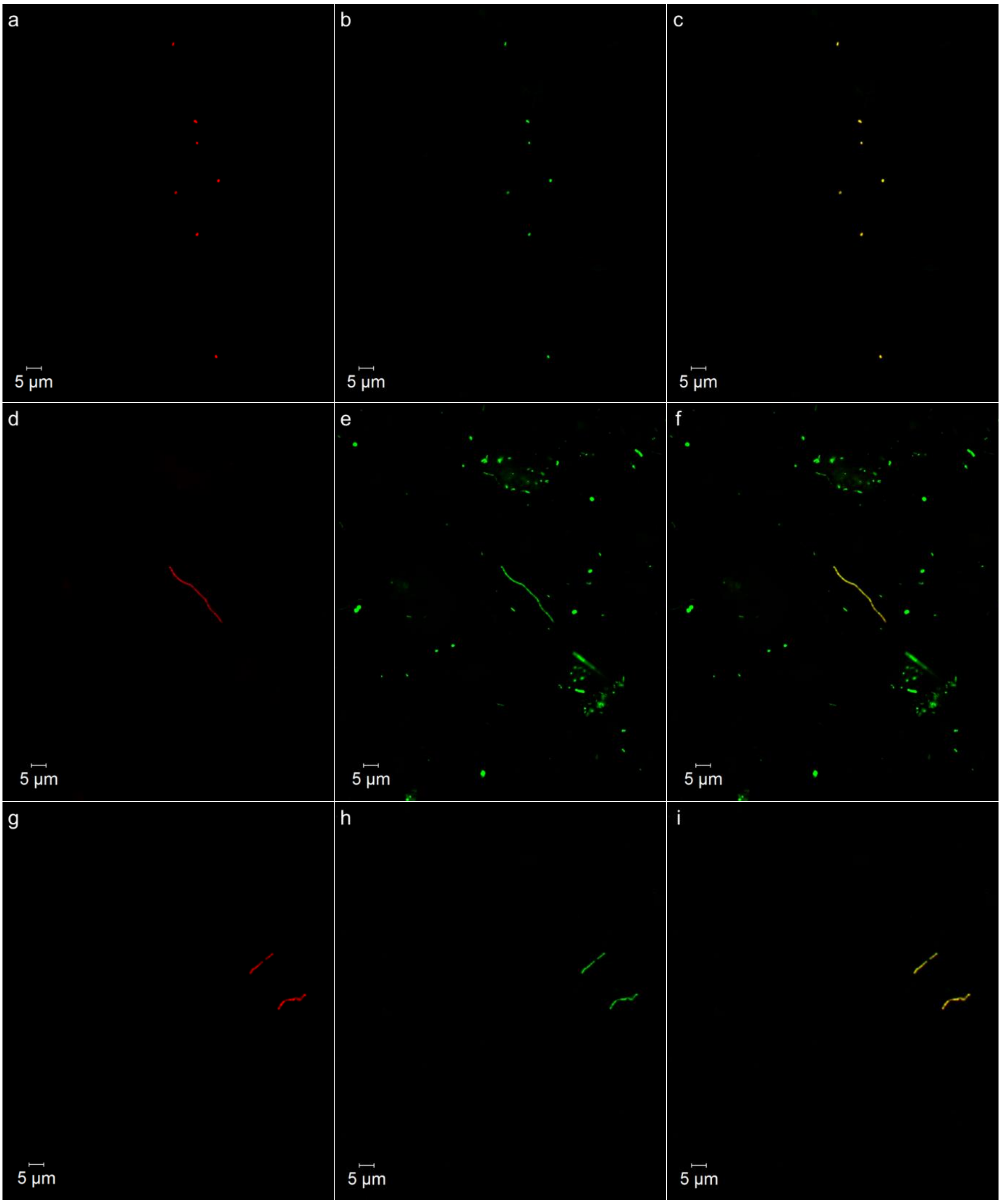

Supplementary Figure 6: Confocal micrographs depicting probe-labelled cells before and after the FACS-sorting process.

a-c: Sorted *Thauera* cells from an activated sludge sample.

d-f: UPWRP_1 cells in a pre-sorted sludge sample that has been broken up by passing the flocs repeatedly through a 26G x ½" syringe needle.

g-i: Sorted UPWRP_1 cells from an activated sludge sample. *Thauera* cells were hybridised with Ribo_Thau1029_17$_{Cy5}$ and EUB338$_{A488}$, whereas UPWRP_1 cells were hybridised with Ribo_Unk1029_17$_{Cy5}$ and EUB338$_{A488}$. Cells were visualised with: (a,d,g) Cy5 filter set (red),

(b,e,h) Alexa488 filter set (green) and (c,f,i) overlap of the two filters (yellow). All figures have a scale bar representing 5 µm, and images were taken with 630x magnification.

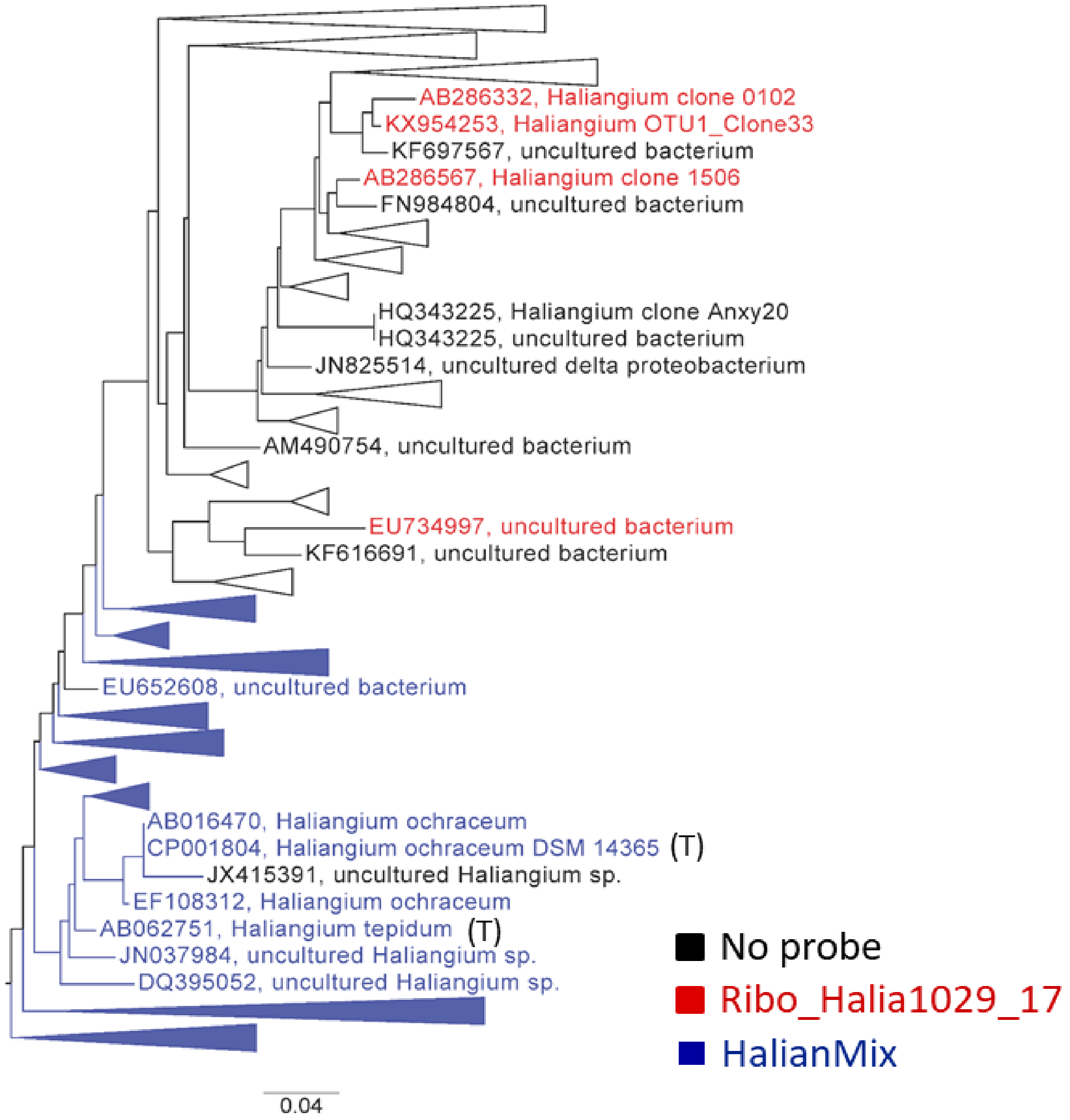

Supplementary Figure 7: 16S rRNA gene sequences of the genus *Haliangium* covered by the various probe combination of Ribo_Halia1029_17 or HalianMix in the ARB-parsimony tree. Legend of the various probes used is located at the bottom right-hand corner of the figure.

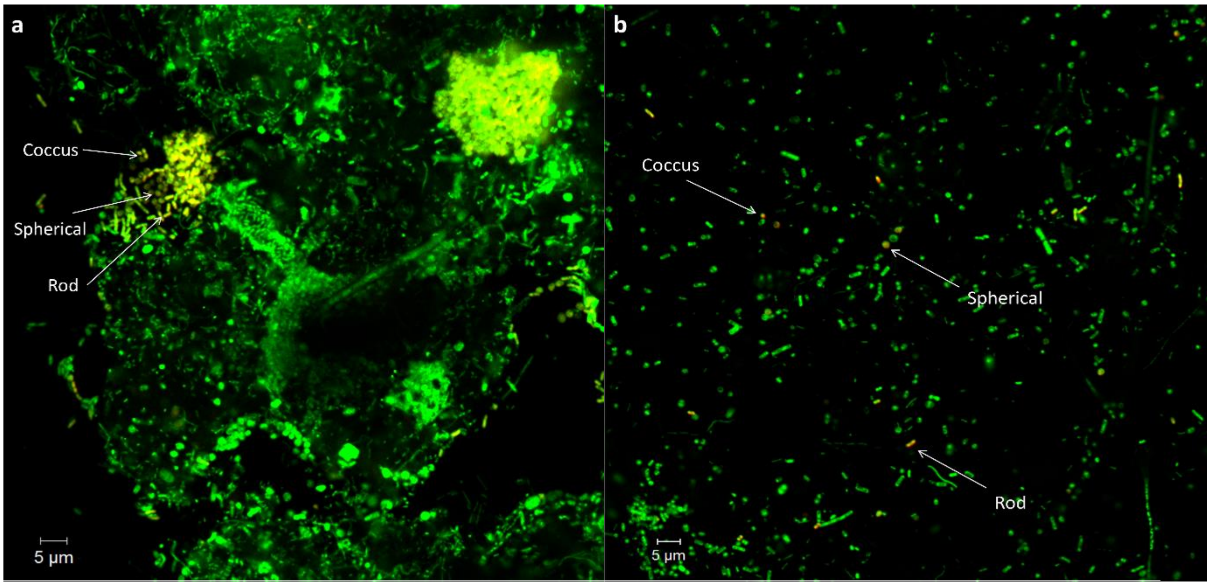

Supplementary Figure 8: Confocal micrographs depicting the different morphotypes of *Haliangium* cells hybridised with probes Ribo_Halia1029_17$_{Cy5}$ (red) and EUB338$_{A488}$ (green) in activated sludge samples. (A) *Haliangium* cells in a fixed and (B) unfixed sample of activated sludge where in-solution FISH protocol was applied. *Haliangium* cells appeared yellow because of the merging of probe signals. Probe-labelled cells with spherical morphology was observed to be larger than the coccus- or rod-shaped cells; the spherical morphology cells had a diameter of up to 2 µm. Arrows demarcate the different morphotypes of *Haliangium*. Bar: 5 µm. Magnification: 63x.

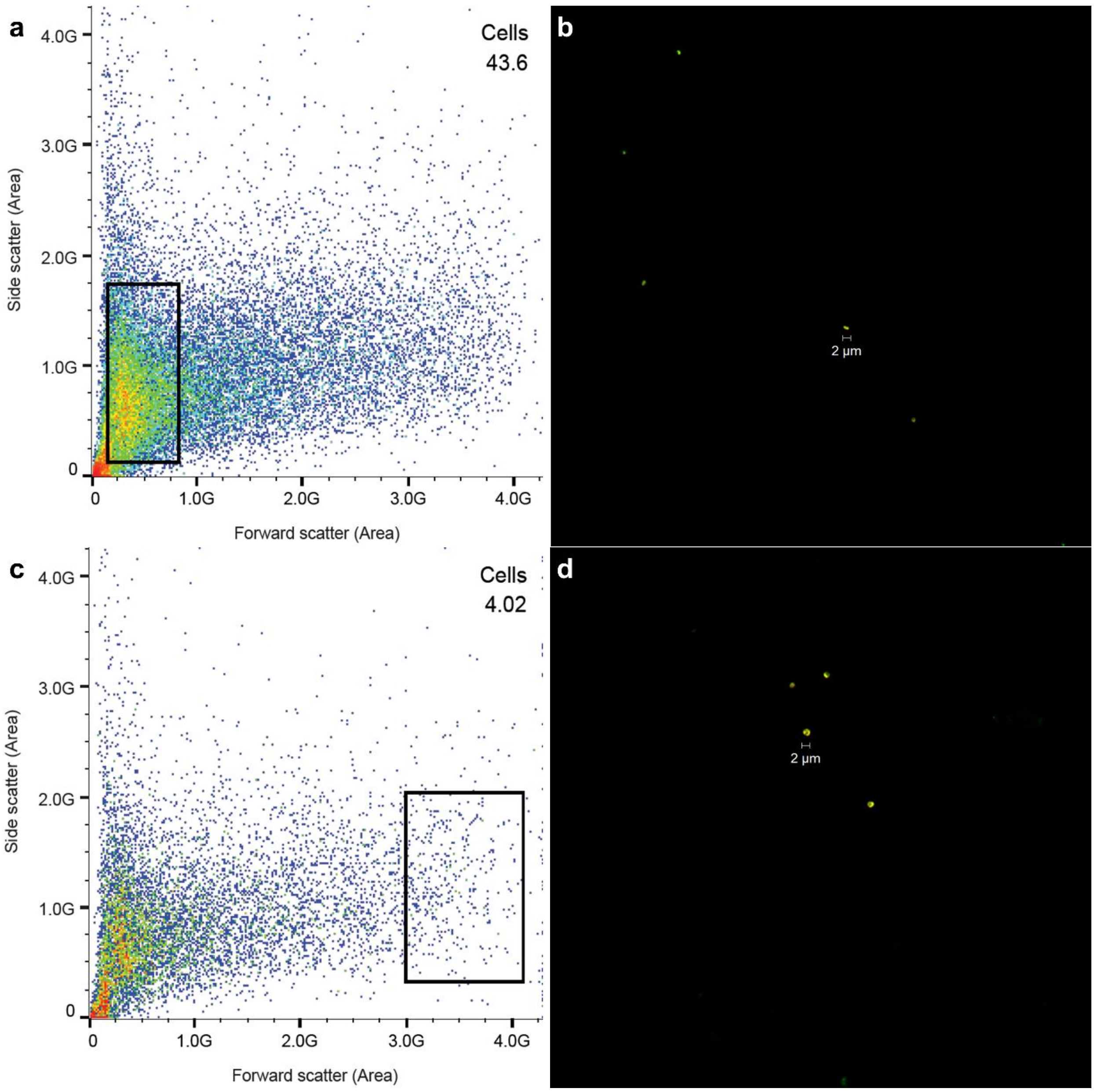

Supplementary Figure 9: Different morphotypes of *Haliangium* cells captured with different sorting gates of forward versus side scatter during the second round of sorting. (A) Sorting of cells with low light-scattering properties led to the collection of (B) rod- and coccus-shaped cells. (C) Sorting of cells with higher light-scattering properties led to the collection of (D) spherical-shaped cells.

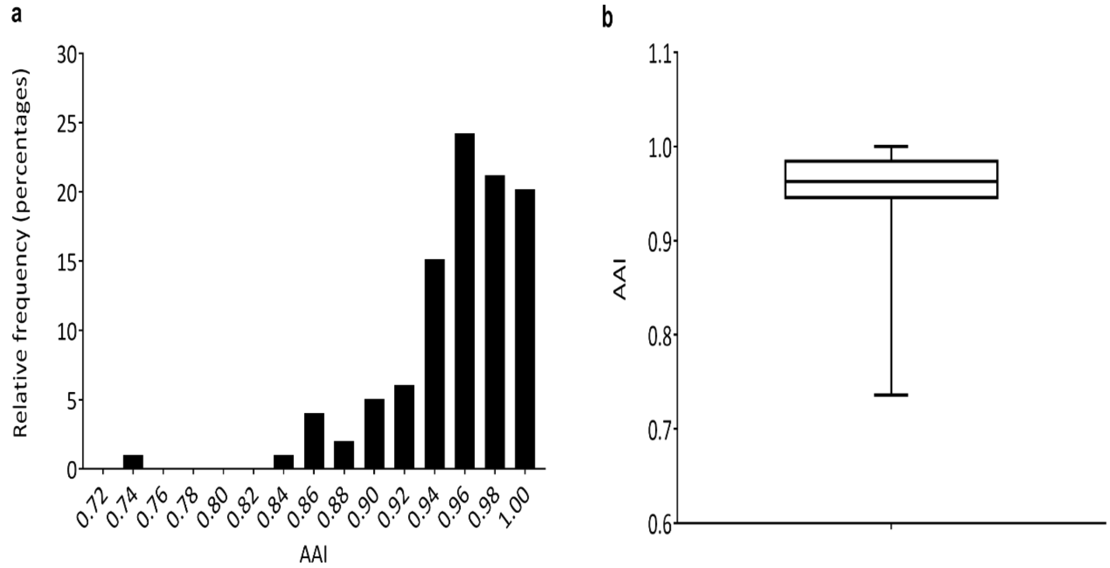

Supplementary Figure 10:

(a) Frequency distribution of the amino acid identity (AAI) of multi-copy lineage-specific marker genes obtained from the draft genome of UPWRP_1.

(b) Box-and-whisker graph depicting the distribution of AAI from multi-copy lineage-specific marker genes obtained from the draft genome of UPWRP_1.

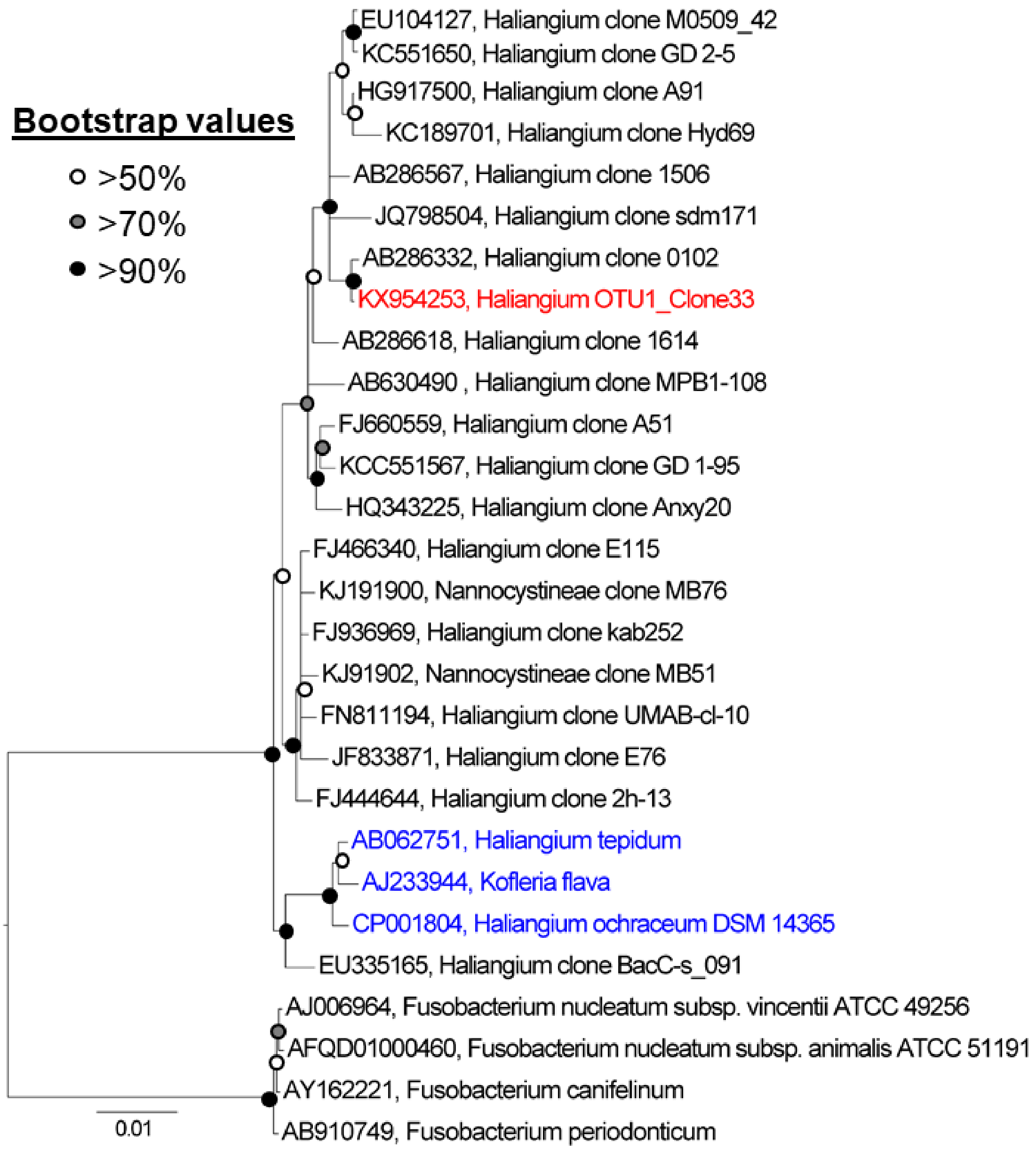

Supplementary Figure 11: Maximum-likelihood (PhyML) phylogenetic tree depicting the 16S rRNA phylogenetic relationship of UPWRP_2 and its closely related sequences in the SILVA database. Members of the genus *Fusobacterium* were used as the outgroup. For 16S rRNA phylogenetic analysis, only near full-length sequences (≥1200 bp) were selected. Representative sequence of OTU is demarcated in red, and sequences of the closest cultured isolates are demarcated in blue. Closely related sequences were obtained from the SILVA 123 SSU Ref NR99 database. Bootstrap values were calculated from 1000 bootstrap analyses and only bootstrap values over 50% were displayed. Branches with low bootstrap values ≤ 50% have been multifurcated. The scale bar represents substitutions per nucleotide base. Legend of the bootstrap values is located at the upper left-hand corner of the diagram.

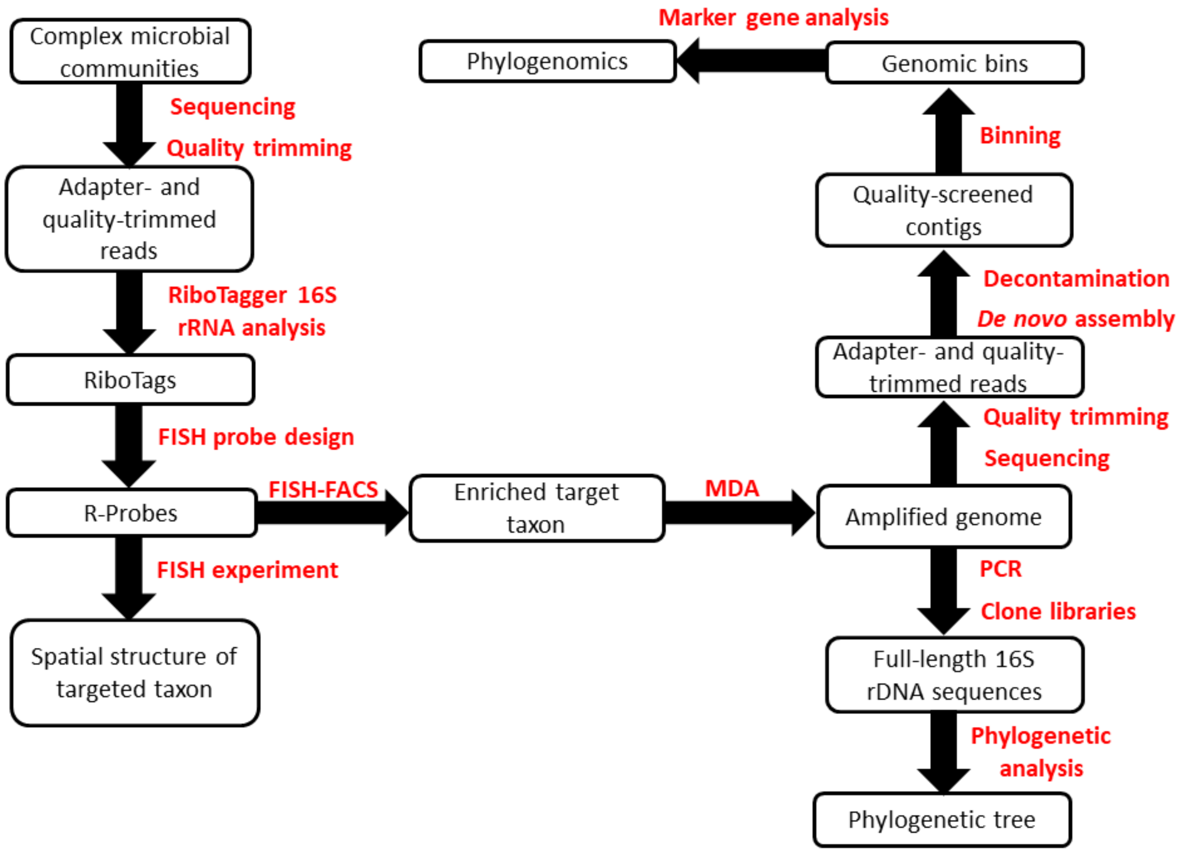

Supplementary Figure 12: Flowchart demonstrating the use of R-Probes for visualisation and genome recovery of targeted microbial groups.
